# Exploring the Causal Association Between 91 Circulating Inflammatory Proteins and Neurodegenerative Diseases: A Bidirectional Two‐Sample Mendelian Randomization and Bioinformatics Analysis

**DOI:** 10.1002/brb3.70586

**Published:** 2025-06-04

**Authors:** Ziwei Gong, Rong Cao, Hong Zhu

**Affiliations:** ^1^ Department of Neurology The Third Xiangya Hospital Central South University Changsha Hunan China; ^2^ Department of Cardiology The Third Xiangya Hospital Central South University Changsha Hunan China; ^3^ Department of Cardiovascular Surgery Xiangya Hospital, Central South University Changsha Hunan China; ^4^ National Clinical Research Center for Geriatric Disorders Xiangya Hospital Central South University Changsha Hunan China

**Keywords:** CD40, circulating inflammatory proteins, Mendelian randomization, multiple sclerosis, neurodegenerative diseases, transcriptome sequencing

## Abstract

**Background:**

Circulating inflammatory proteins play a significant role in the pathogenesis of neurodegenerative diseases (NDDs). However, the precise causal relationship and the underlying mechanisms of their interaction remain elusive.

**Methods:**

Genome‐wide association study (GWAS) data for 91 circulating inflammatory proteins were obtained from the GWAS Catalog. Additionally, GWAS data for Parkinson's disease (PD), Alzheimer's disease (AD), amyotrophic lateral sclerosis (ALS), multiple sclerosis (MS), and ischemic stroke (IS) were acquired from the IEU Open GWAS Project. Four Mendelian randomization (MR) methods were employed to analyze causal effects, accompanied by sensitivity and pleiotropy analyses. Expression quantitative trait loci (eQTL) analyses for CD40 and MS‐associated SNPs were performed. Transcriptomic data from the peripheral blood of MS patients were used to identify differentially expressed genes (DEGs) in relapsing‐remitting MS (RRMS). RRMS patients were divided into two subgroups (C1 and C2) based on CD40 expression levels for comparative analysis. A single gene set enrichment analysis (GSEA) was conducted to investigate potential molecular mechanisms through which CD40 influences MS.

**Results:**

MR analyses indicated that CD40 ligand receptor (CD40) is associated with a reduced risk of MS (OR, 0.78; 95% CI, 0.72–0.84; *P*_FDR_ = 8.75E‐07). No statistically significant bidirectional causal relationships were found between other inflammatory proteins and PD, AD, ALS, or IS, and the findings were robust. Functional enrichment analysis revealed that these eQTLs primarily relate to transcriptional regulation, herpes simplex virus 1 (HSV‐1) infection, and bile and fatty acid metabolism. In MS peripheral blood microarray data, CD40 is significantly downregulated in RRMS. Intergroup comparisons revealed elevated levels of resting memory CD4^+^ T cells, activated NK cells, and neutrophils in C1, alongside increased autophagy, apoptosis, multiple immune responses, and upregulation of transforming growth factor‐β (TGF‐β) signaling pathways. Conversely, C2 exhibited higher levels of Tregs, resting NK cells, and activated dendritic cells, as well as upregulation in processes such as cholesterol homeostasis, glucose metabolism, and CD4/CD8 downregulation. Single‐GSEA results suggest that CD40 promotes nucleotide metabolism, mitochondrial calcium ion transport, unfolded protein response (UPR), and adaptive immune regulation, while inhibiting androgen response and TGF‐β signaling pathways, thereby influencing the progression of RRMS.

**Conclusion:**

CD40 may exert neuroprotective effects in MS patients via diverse cellular and molecular pathways, potentially representing a novel target for MS intervention.

## Graphical abstract

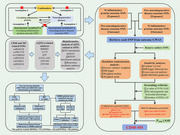

**Abbreviations**
AD: Alzheimer's disease
ADT: androgen deprivation therapy
ALS: amyotrophic lateral sclerosis
BP: biological processes
CD40: CD40 ligand receptor
CDF: cumulative distribution function
CNS: central nervous system
CXCL8: chemokines like CXC motif chemokine ligand 8
EAE: experimental autoimmune encephalitis
EndoMT: endothelial–mesenchymal transition
eQTL: expression quantitative trait loci
ES: enrichment score
FDR: false discovery rate
FGF‐21: fibroblast growth factor 21
FLT‐3L: Fms‐related tyrosine kinase 3 ligand
GO: Gene Ontology
GSEA: gene set enrichment analysis
GWASs: genome‐wide association studies
HSV‐1: Herpes simplex virus 1
IL‐1β: interleukin‐1β
IS: ischemic stroke
IVs: instrumental variables
IVW: inverse variance‐weighted
KEGG: Kyoto Encyclopedia of Genes and Genomes
LD: linkage disequilibrium
LIF receptor: leukemia inhibitory factor receptor
MF: molecular functions
MHC: major histocompatibility complex
MMP‐1: matrix metalloproteinase‐1
MOG: myelin oligodendrocyte glycoprotein
MR: Mendelian randomization
MR‐PRESSO: MR Pleiotropy Residual Sum and Outlier
MS: multiple sclerosis
NDDs: neurodegenerative disorders
OPG: osteoprotegerin
PD: Parkinson's disease
PPMS: primary progressive multiple sclerosis
pQTL: quantitative trait loci
RRMS: relapsing‐remitting multiple sclerosis
SCF: stem cell factor
SNPs: single‐nucleotide polymorphisms
SPMS: secondary progressive multiple sclerosis
TGF‐α: transforming growth factor‐alpha
TMEV: Theiler's murine encephalitis virus
TNFRSF9: tumor necrosis factor receptor superfamily member 9
UPR: unfolded protein response

## 1 Introduction

Neurodegenerative disorders (NDDs) encompass a spectrum of age‐related, heterogeneous neurological conditions characterized by the progressive loss of neurons and continuous disruption of neural networks, leading to extensive impairments in memory, cognition, sensation, movement, and behavior (Wilson et al. 2023; Agnello and Ciaccio 2022). Common NDDs, such as Parkinson's disease (PD), Alzheimer's disease (AD), amyotrophic lateral sclerosis (ALS), multiple sclerosis (MS), and ischemic stroke (IS), exhibit high incidence and disability rates, imposing significant economic and social burdens globally (Wilson et al. 2023, Logroscino et al. 2022; 2023 Alzheimer's Disease Facts and Figures 2023; Tolosa et al. 2021; Feldman et al. 2022; Dobson and Giovannoni 2019; Johnson et al. 2016). Given the unclear pathogenesis of these disorders, the suboptimal effectiveness of current treatments, and the large affected population, elucidating their pathogenic factors and treatment targets is critical.

An increasing body of evidence suggests that inflammation is associated with various diseases, including cardiovascular diseases, kidney diseases, and NDDs (Speer et al. 2022; Kwon and Koh 2020). The activation of microglia and astrocytes, critical regulators in the neuroinflammatory response, serves as a pathological hallmark for NDDs, including AD, PD, ALS, MS, and IS (Kwon and Koh 2020; Serrano‐Pozo et al. 2011; McGeer and McGeer 2008; Philips and Robberecht 2011; Zelic et al. 2021; Jurcau and Simion 2021). Circulating inflammatory proteins such as cytokines, growth factors, and chemokines play pivotal roles as signaling molecules, influencing the progression and prognosis of neuronal degeneration in NDDs. For instance, numerous observational studies and meta‐analyses have demonstrated elevated levels of pro‐inflammatory cytokines, including interleukin‐1 β (IL‐1β), IL‐2, IL‐6, IL‐8, and IL‐17 (Veryard et al. 2012; Hu et al. 2017; Qin et al. 2016; X. Chen et al. 2018; Yi et al. 2024), and chemokines like CXC motif chemokine ligand 8 (CXCL8) and CXCL13 (Bai et al. 2019; Lawlor et al. 2008) in the peripheral blood of patients with various NDDs. These biomarkers may also serve as therapeutic targets. Therefore, elucidating the relationship between circulating inflammatory proteins and NDDs could facilitate the development of more targeted prevention and treatment strategies.

Mendelian Randomization (MR) analysis is a genetic epidemiological method that adheres to Mendelian inheritance laws and employs single‐nucleotide polymorphisms (SNPs) from large‐scale population data in genome‐wide association studies (GWASs) as instrumental variables (IVs) for causal inference (Emdin et al. 2017). Consequently, this study aims to (1) investigate the bidirectional causal relationship between 91 circulating inflammatory proteins and five NDDs; (2) conduct expression quantitative trait loci (eQTL)‐related analyses on SNPs that show positive results to preliminarily explore the mechanisms by which circulating inflammatory proteins influence NDDs; and (3) explore the peripheral blood transcriptome data of NDD patients to further elucidate the cellular and molecular mechanisms underlying these connections and validate MR results using bioinformatics methods.

## 2 Materials and Methods

### 2.1 Research Design

**FIGURE 1:**
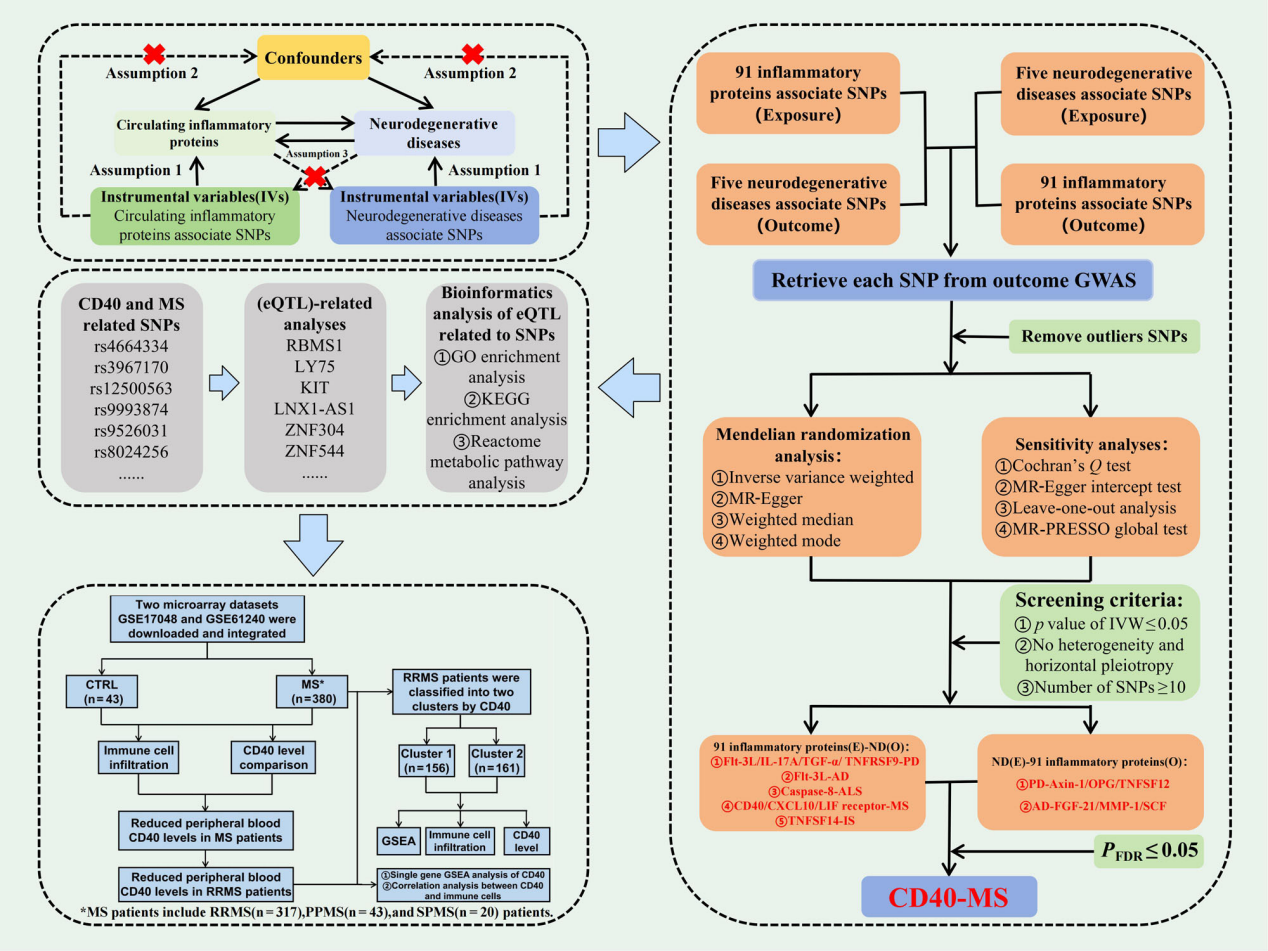
Detailed flowchart of this study.

MR analysis evaluates the bidirectional causal relationships between 91 circulating inflammatory proteins and NDDs, contingent upon three critical hypotheses: (1) correlation hypothesis: SNPs must be strongly correlated with the exposure; (2) independence hypothesis: SNPs should not be associated with any potential confounding factors; (3) exclusivity hypothesis: SNPs affect outcomes solely through exposure. Following rigorous quality control and the removal of confounding factors, we performed eQTL‐related analysis, functional enrichment analysis, and protein–protein interaction (PPI) network construction focusing on CD40 and MS‐related SNPs. Subsequently, a series of bioinformatics analyses were conducted on the peripheral blood microarray data of MS patients to validate the MR findings. The flowchart of this study is detailed in Figure 1. The cohort is comprised solely of European populations, all analysis data are publicly accessible, and the original study has received ethical approval. MR research adheres to the STROBE‐MR guidelines (Skrivankova et al. 2021).

### 2.2 Data Sources of MR Analysis

The summary data for 91 circulating inflammatory proteins were obtained from the GWAS Catalog (https://www.ebi.ac.uk/gwas/). This study utilized the Olink Target Inflammation panel to measure these proteins in plasma samples from 14,824 individuals of European ancestry across 11 cohorts and conducted a genome‐wide quantitative trait loci (pQTL) study (Zhao et al. 2023). GWAS data for five NDDs were sourced from the IEU Open GWAS Project (https://gwas.mrcieu.ac.uk/): PD data from the International Parkinson's Disease Genomics Consortium included 33,674 patients and 449,056 controls (Nalls et al. 2019). AD data from the International Genomics of Alzheimer's Project included 21,982 patients and 41,944 controls (Kunkle et al. 2019). ALS data from the International Amyotrophic Lateral Sclerosis Genomics Consortium comprised 12,577 patients and 23,475 controls (Van Rheenen et al. 2016). MS data from the International Multiple Sclerosis Genetics Consortium included 47,429 patients and 68,374 controls (International Multiple Sclerosis Genetics Consortium 2019). IS data from the International Stroke Genetics Consortium included 10,307 patients and 19,326 controls (Malik et al. 2016). Detailed information regarding these datasets is available in Table 1.

**TABLE 1:** Summary of the genome‐wide association study dataset in this study.

Trait	GWAS ID	Database	Population	Ncase	Ncontrol	Sample size	Number of SNPs
Parkinson's disease	ieu‐b‐7	IEU open	European	33,674	449,056	482,730	17,891,936
Alzheimer's disease	ieu‐b‐2	IEU open	European	21,982	41,944	63,926	10,528,610
Amyotrophic lateral sclerosis	ieu‐a‐1085	IEU open	European	12,577	23,475	36,052	7,740,345
Multiple sclerosis	ieu‐b‐18	IEU open	European	47,429	68,374	115,803	6,304,359
Ischemic stroke	ieu‐a‐1108	IEU open	European	10,307	19,326	29,633	2,421,920
91 Circulating inflammatory proteins	GCST90274758‐GCST90274848	GWAS Catalog	European	N/A	N/A	14,824	2868(clump‐*R* ^2^ < 0.001, clump‐kb < 10,000, *p* < 5E‐06)

Abbreviation: N/A, not applicable.

### 2.3 Selection of Instruments

Initially, SNPs significantly associated with circulating inflammatory proteins or NDDs were selected as IVs. When analyzing the influence of 91 inflammatory proteins, we initially set the *p* < 5 × 10^−8^. However, this threshold yielded an insufficient number of qualifying SNPs. To ensure a robust sample size, we adjusted the threshold to *p* < 5 × 10^−6^, a common practice in many MR studies on NDDs (Cullell et al. 2021; Cui et al. 2022, Yin et al. 2023). For reverse MR analyses, the more stringent *p* < 5 × 10^−8^ was maintained. Secondly, we performed a linkage disequilibrium (LD) test, clustering genetic variations with an *R* ^2^ > 0.001 and a window size of 10,000 kb, to exclude SNPs with palindromic sequences, intermediate allele frequencies, or those closely related to outcomes. Thirdly, we assessed the strength of association between IVs and exposure by calculating the *F*‐statistics. Specifically, for each SNP, its *F*‐statistic was calculated as *F* = Beta^2^/SE^2^, where Beta denotes the effect size of the SNP on the exposure, and SE is the corresponding standard error. An *F*‐statistic > 10 indicates that there is a strong correlation between IVs and exposure, which can effectively avoid the influence of weak IV bias on the results of MR analysis (Burgess et al. 2011). The PhenoScanner database (http://www.phenoscanner.medschl.cam.ac.uk) was utilized to exclude IVs with confounding traits. All relevant SNPs are listed in Tables S9 and S10.

### 2.4 Bidirectional Two‐Sample MR Analysis

The inverse variance‐weighted (IVW) method was employed to evaluate the causal relationship between inflammatory proteins and NDDs, supplemented by MR‐Egger, weighted median, and weighted mode methods. IVW estimates causal effects by integrating weighted Wald ratios (exposure–outcome effect estimates divided by SNP‐exposure effect estimates) across all SNPs. Each SNP's weight corresponds to the inverse variance of its effect estimate, giving greater weight to more precise SNPs. By combining these weighted effects, IVW provides robust causal estimates while strictly adhering to MR's three critical hypotheses. This weighting approach effectively minimizes random error and enhances statistical power (Pierce and Burgess 2013; Burgess et al. 2013). MR‐Egger addresses pleiotropic bias through an intercept‐based regression model. This approach regresses outcome effects against exposure effects across all SNPs, where the regression slope represents the corrected causal effect and the intercept quantifies directional pleiotropy (SNP effects not mediated by exposure). This method offers the distinct advantage of quantifying bias magnitude and is routinely employed as a complementary approach to IVW for pleiotropy assessment and sensitivity analyses (Bowden, Del Greco M, et al. 2016; Burgess and Thompson 2017). Weighted median derives causal effect estimates by computing a weighted median of genetic variant associations. When at least 50% of the IVs satisfy the MR assumptions, the method maintains robust causal estimates despite heterogeneity among the remaining SNPs. The method yields intuitive and readily interpretable results (Bowden, Davey Smith, et al. 2016). Weighted mode accommodates pleiotropy among IVs, operating under the core assumption that the majority of IVs exhibit zero pleiotropy (affecting the outcome exclusively through the exposure). Even when some SNPs demonstrate pleiotropic effects, this approach maintains stable causal effect estimation (Hartwig et al. 2017).

This study implemented multiple sensitivity analyses to validate the reliability of the results. Cochran's *Q*‐test assesses heterogeneity among IVs under the null hypothesis that all IVs share identical effect sizes (indicating no heterogeneity). Rejection of the null hypothesis (*p *< 0.05) provides evidence of significant heterogeneity in the effect estimates (Del Greco M et al. 2015). The MR‐Egger intercept test assesses horizontal pleiotropy by examining the intercept deviation from zero. A statistically significant intercept (*p *< 0.05) suggests that SNPs influence outcomes through non‐exposure pathways, violating MR's core assumptions (Hemani et al. 2018). MR‐PRESSO identifies and corrects horizontal pleiotropy by comparing observed effect estimates and simulated distributions after removing outlier SNPs. This approach eliminates abnormal SNPs to minimize bias in causal effect estimation (Verbanck et al. 2018). Leave‐one‐out analysis examines the potential impact of abnormal IVs by sequentially removing each SNP. When effect estimates remain consistent across all iterations, this demonstrates the result's reliability and confirms that there is no excessive influence of a single SNP (Gu et al. 2024).

The criteria for filtering MR results included (1) a *p* value of IVW < 0.05, (2) no heterogeneity and horizontal pleiotropy, and (3) a number of SNPs ≥ 10. Furthermore, the statistical significance of the causal relationship was determined using the False Discovery Rate (FDR) method within the Bonferroni correction, targeting a *P*_FDR_ < 0.05. MR analysis was performed using the TwoSampleMR package (version 0.5.7) in R software (version 4.3.1).

### 2.5 The eQTL‐Related Analysis of SNPs

CD40 and MS‐related SNPs were searched in the QTL database (http://www.mulinlab.org/qtlbase/index.html) for eQTL with a *p* < 0.05, restricting the tissue type to the brain (Zheng et al. 2020). Gene Ontology (GO), Kyoto Encyclopedia of Genes and Genomes (KEGG) enrichment analyses, and Reactome metabolic pathway analysis were conducted using the DAVID (https://david.ncifcrf.gov/) and Reactome (https://reactome.org/) databases. The GO analysis involved functional annotation of eQTL‐mediated biological processes (BP) and molecular functions (MF). Statistical significance was determined by a *p* < 0.05 or *P*_FDR_ < 0.05. The STRING database (https://cn.string‐db.org/) was utilized to construct PPI networks.

### 2.6 Microarray Data of Transcriptomic Analyses

From the GEO database (https://www.ncbi.nlm.nih.gov/geo/), we obtained two microarray datasets related to MS: GSE17048 and GSE61240. The GSE17048 dataset (GPL6947 platform) comprises data from 45 healthy individuals and 99 MS patients, including 43 with primary progressive MS (PPMS), 36 with relapsing‐remitting MS (RRMS), and 20 with secondary progressive MS (SPMS). The GSE61240 dataset (GPL570 platform) includes data from 550 RRMS patients.

### 2.7 Data Processing and Identification of DEGs

We utilized the R package “inSilicoMerging” to merge and normalize the dataset, followed by the use of the BioConductor “SVA” package to remove batch effects (Vancamelbeke et al. 2017). The “limma” R package was employed to identify DEGs, with the threshold |log2(Fold Change)|>1.5 and *p* < 0.05.

### 2.8 Cluster Analysis of RRMS Based on CD40 Expression Level and Intergroup Comparison

Consensus clustering analysis was conducted on 586 RRMS samples based on CD40 expression levels using the R package “ConsensusClusterPlus.” The optimal number of clusters was determined based on the cumulative distribution function (CDF) curve and sample clustering consistency score. The CIBERSORT algorithm (https://cibersort.stanford.edu/) was used to assess the infiltration abundance of 22 immune cells across RRMS subgroups. The Pearson correlation coefficient was used to analyze the correlation between CD40 and various immune cells, with a threshold of *p* < 0.05. Gene set enrichment analysis (GSEA) was performed using three software packages from the MSigDB database (http://www.gsea‐msigdb.org/gsea/downloads.jsp) to evaluate the functional differences between CD40‐related RRMS subgroups: “c2.cp.kegg.v7.4.symbols.gmt,” “c2.cp.reactome.v7.4.symbols.gmt,” and “h.all.v7.4.symbols.gmt.”

### 2.9 GSEA of CD40

GSEA software (version 3.0) was obtained from the GSEA website (http://software.broadinstitute.org/gsea/index.jsp) and divided the samples into two groups based on CD40 expression levels: a high expression group (≥ 50%) and a low expression group (< 50%). Additionally, we used the three MSigDB software packages from the previous section to evaluate the relevant pathways and molecular mechanisms.

### 2.10 Statistical Analysis

R software (version 4.0.3) was applied to perform statistical analyses. The Pearson correlation coefficient was utilized to analyze the correlation between CD40 and immune cells. In MR studies, a *p* < 0.05 represents nominal significance, while *P*_FDR_ < 0.05 represents statistical significance. In eQTL and bioinformatics analyses, both *p* < 0.05 and *P*_FDR_ < 0.05 are considered statistically significant.

## 3 Results

### 3.1 The Causal Relationship Between 91 Circulating Inflammatory Proteins and NDDs

**FIGURE 2:**
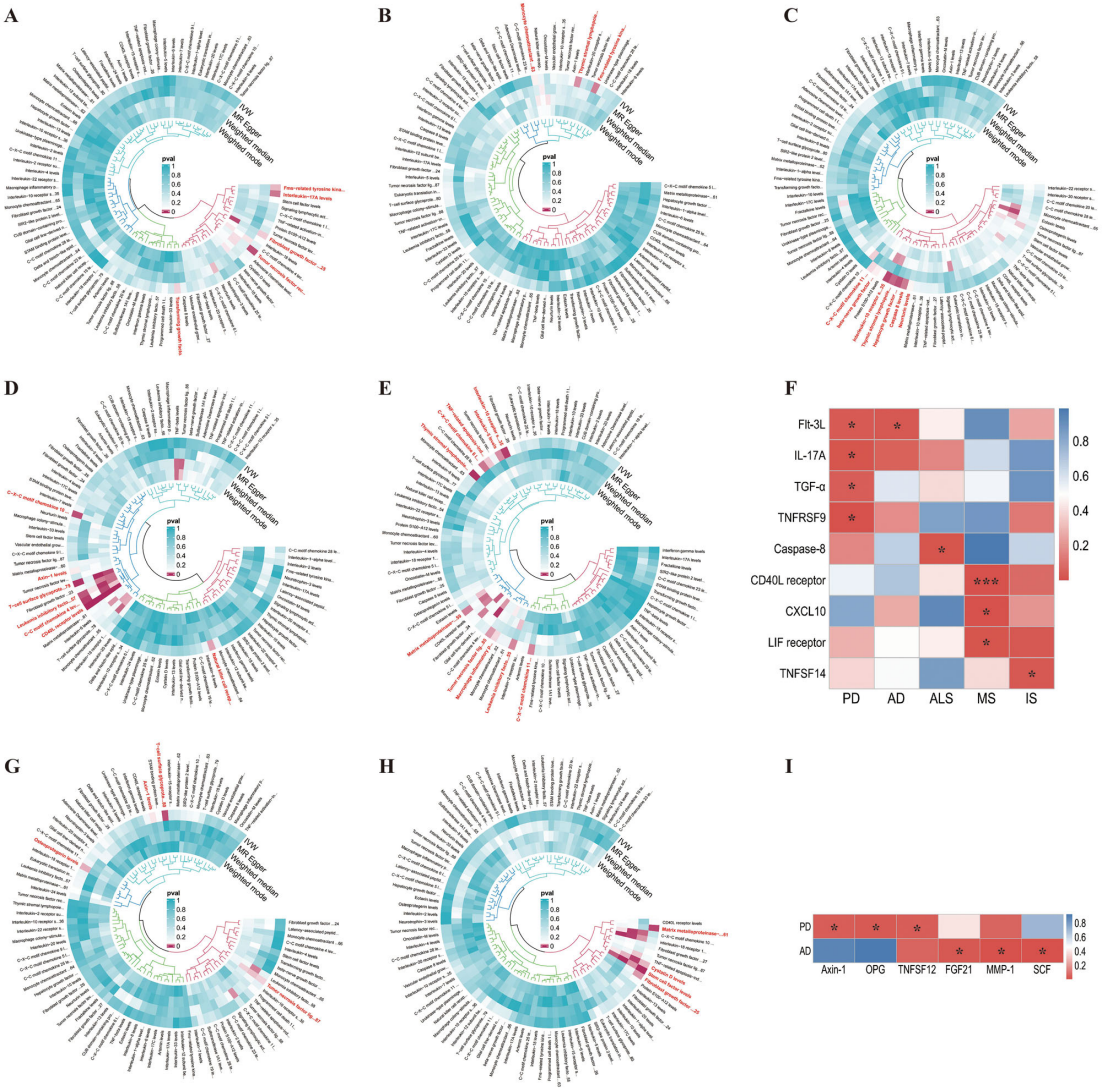
Bidirectional causal relationship between 91 circulating inflammatory proteins and NDDs. (A–E) MR analysis results for circulating inflammatory proteins and PD (A), AD (B), ALS (C), MS (D), and IS (E). (G–H) Reverse MR analysis results for PD (G) and AD (H) with circulating inflammatory proteins. (F, I) Heat maps summarizing positive results in bidirectional MR analysis. *p < 0.05, ***P_FDR_ < 0.05.

**FIGURE 3:**
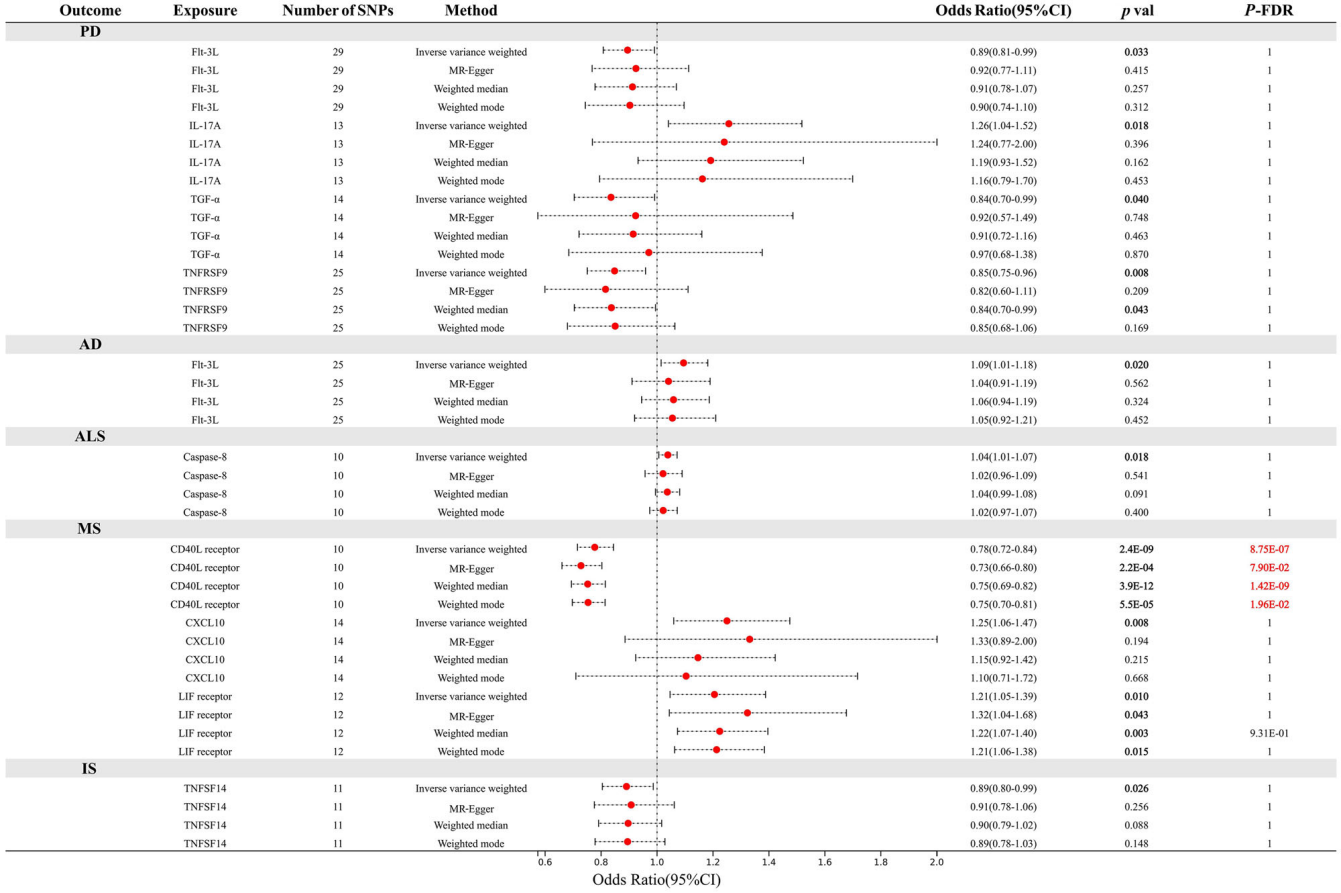
The relationship between circulating inflammatory proteins and the risk of five types of NDDs. *p *< 0.05 represents a nominally causal association, while *P*_FDR_ < 0.05 represents a statistically causal association.

The names of 91 circulating inflammatory proteins are summarized in Table S1, and the MR analysis results between these proteins and PD (Figure 2A), AD (Figure 2B), ALS (Figure 2C), MS (Figure 2D), and IS (Figure 2E) are depicted using a circular heatmap. After screening, Fms‐related tyrosine kinase 3 ligand (FLT‐3L), transforming growth factor‐alpha (TGF‐α), and tumor necrosis factor receptor superfamily member 9 (TNFRSF9) are associated with a low risk of PD, while IL‐17A is associated with a high risk of PD. FLT‐3L is associated with a high risk of AD, while caspase‐8 is associated with a high risk of ALS. The CD40 ligand receptor (CD40) is associated with a low risk of MS, whereas CXCL10 and the leukemia inhibitory factor receptor (LIF receptor) are associated with a high risk of MS. TNFSF14 is associated with a low risk of IS. After FDR correction, only CD40 was negatively correlated with the risk of MS (OR: 0.78; 95% CI, 0.72–0.84; *P*_FDR_ = 8.75E‐07). The heatmap (Figure 2F) and forest plot (Figure 3) illustrate the causal relationship between circulating inflammatory proteins and NDDs. Detailed information on the MR analysis results and the SNPs used can be found in Tables S2–S9.

### 3.2 The Causal Relationship Between NDDs and 91 Circulating Inflammatory Proteins

**FIGURE 4:**
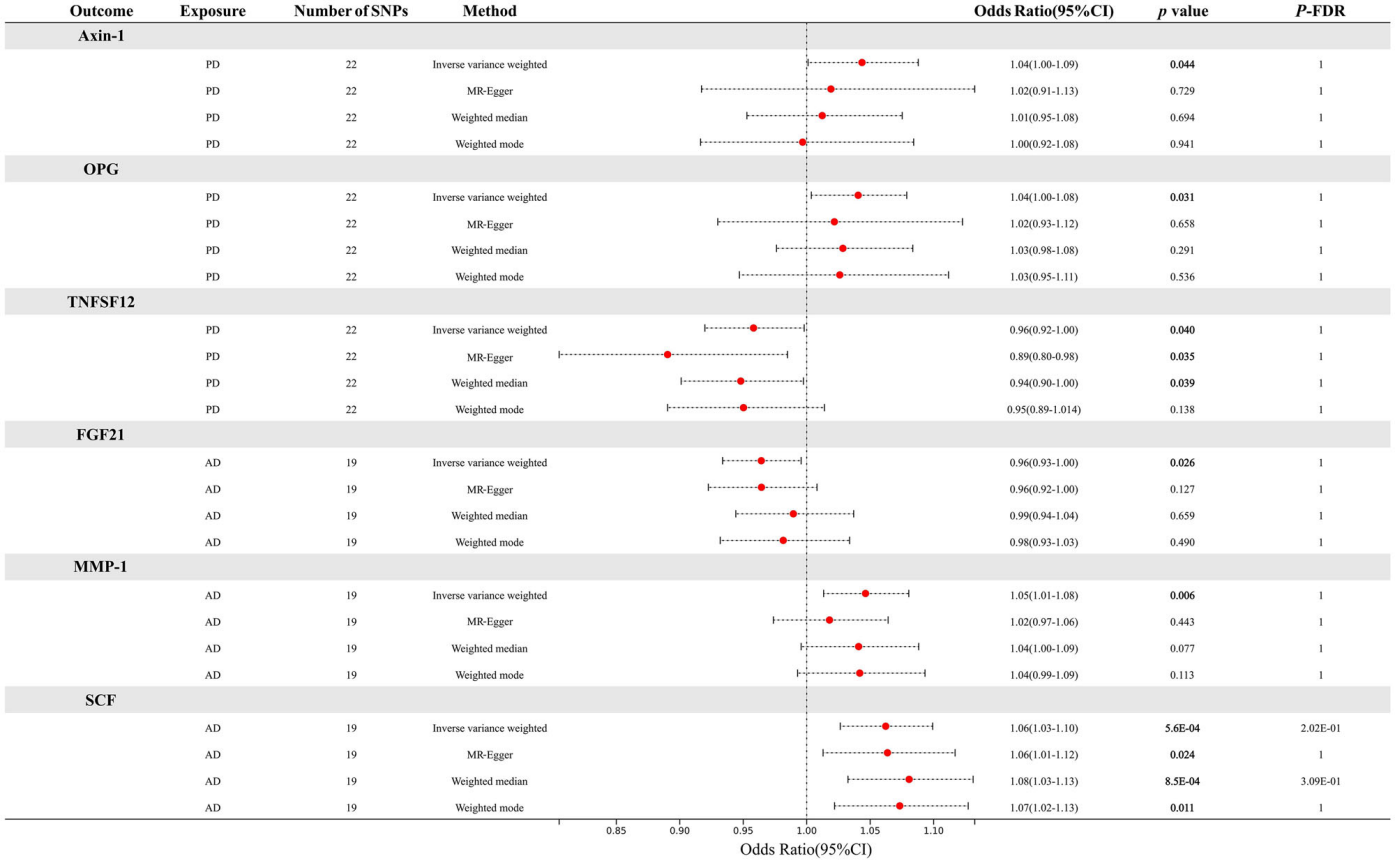
The relationship between PD, AD, and levels of circulating inflammatory proteins. *p *< 0.05 represents nominally causal association, while *P*_FDR_ < 0.05 represents a statistically causal association.

The reverse MR analysis results showed a nominally causal association between PD and Axin‐1, osteoprotegerin (OPG), and TNFSF12 (Figure 2G,I). AD has a nominally causal association with fibroblast growth factor 21 (FGF‐21), matrix metalloproteinase‐1 (MMP‐1), and stem cell factor (SCF) (Figure 2H,I). After FDR correction, there is no statistically significant causal correlation between NDDs and circulating inflammatory proteins, as shown in the forest plot (Figure 4). The results of the reverse MR analysis and detailed information on the SNPs used are shown in Table S10.

### 3.3 Sensitivity and Horizontal Pleiotropy Analysis of Bidirectional MR Results

Cochran's *Q* analysis showed no heterogeneity in the results of the bidirectional MR analysis (all *p* > 0.05, Table S11). Similarly, the MR‐Egger intercept test and MR‐PRESSO analysis did not indicate horizontal pleiotropy (all *p* > 0.05, Table S11). The scatter plot, funnel plot, and leave‐one‐out plot further demonstrate the reliability of the bidirectional MR results (Figures S1–S7).

### 3.4 The eQTL Analysis of CD40 and MS‐Related SNPs

**FIGURE 5:**
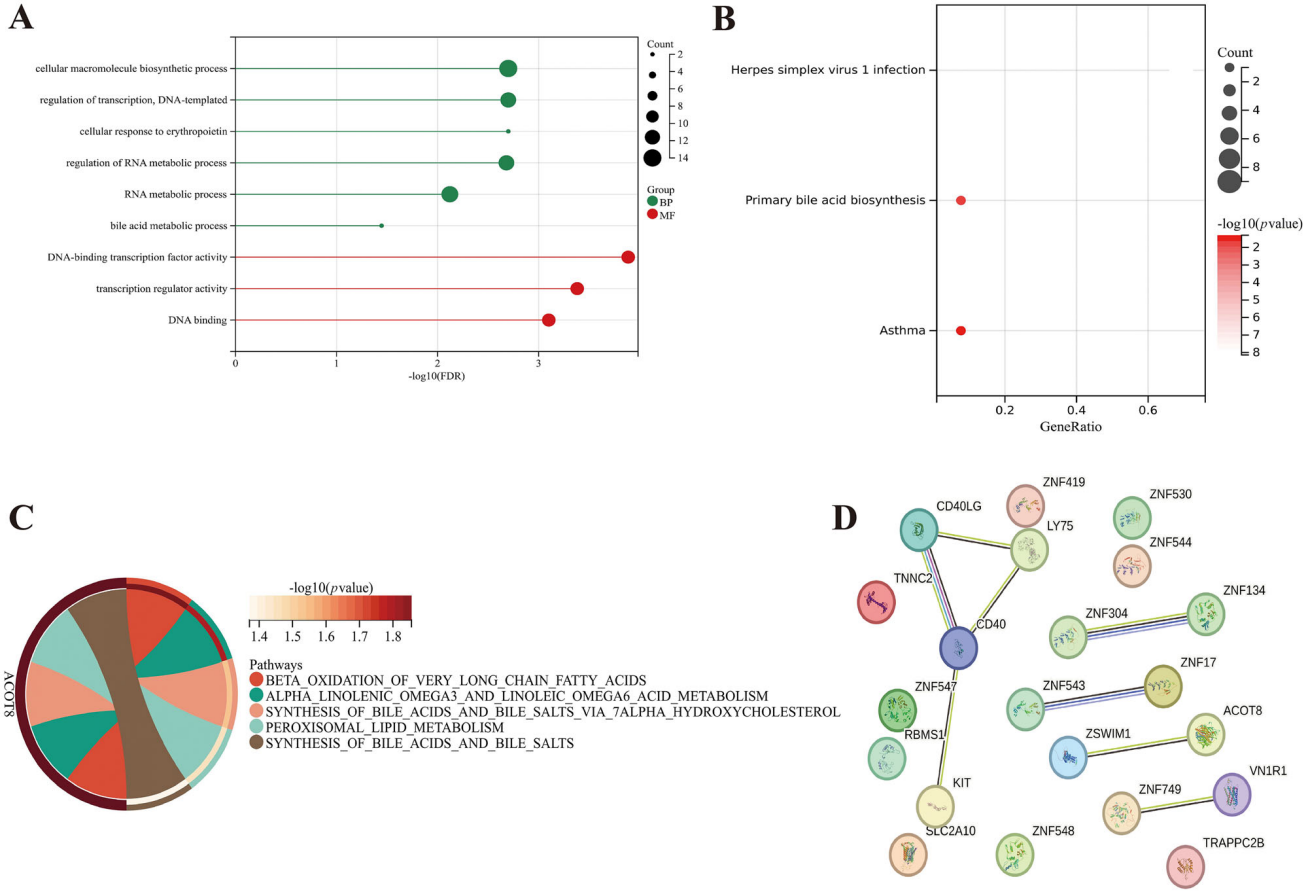
The eQTL‐related analysis of CD40 and MS‐related SNPs. GO (A), KEGG (B), and Reactome metabolic pathway (C) enrichment analysis of eQTL, as well as PPI network (D).

In the MR analysis of CD40 and MS, we included 10 SNPs. Among these, 21 eQTLs were closely related to 4 SNPs after removing duplicates (Table S12). Functional enrichment analysis showed that these eQTLs are mainly involved in the regulation of transcription, cellular response to erythropoietin, RNA and bile acid metabolic processes, and DNA binding (Figure 5A). They are also related to herpes simplex virus 1 (HSV‐1) infection, primary bile acid biosynthesis, and asthma (Figure 5B). Additionally, these eQTLs participate in processes such as fatty acid metabolism, bile acid, and bile salt biosynthesis (Figure 5C). Among the 21 eQTLs, 12 have interactions with each other (Figure 5D).

### 3.5 Identification of DEGs Related to MS Patients

**FIGURE 6:**
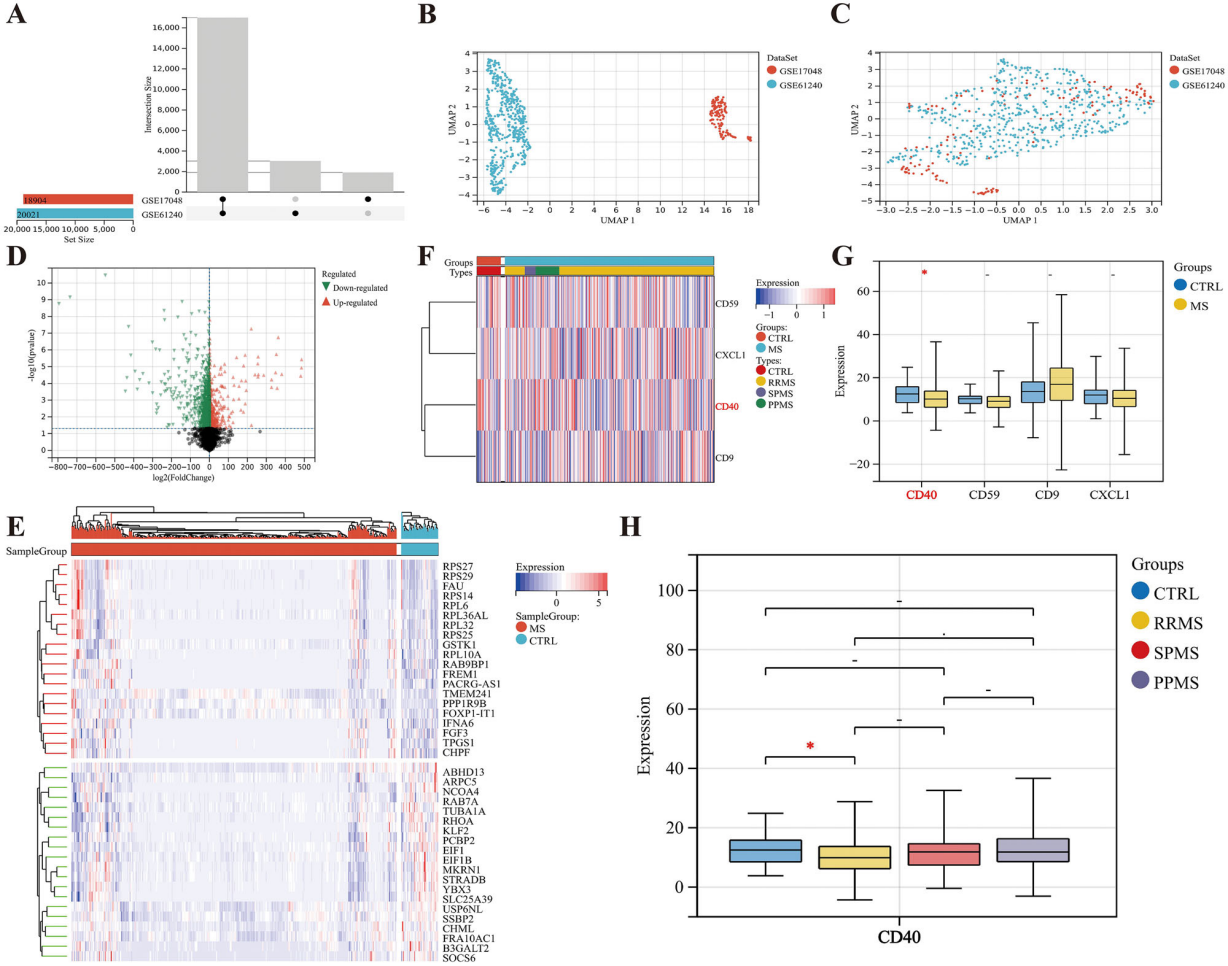
Processing of peripheral blood microarray data and identification of DEGs in MS patients. (A–C) Normalization of two microarray datasets (A) and UMAP plot results before and after removing batch effects (B, C). (D) Volcano plots of DEGs. (E) Heatmaps of the 20 most significantly upregulated and downregulated DEGs. (F–H) Expression levels of CD40 between the healthy individuals and MS and its three subtypes are displayed using heatmaps (F) and box plots (G, H). “‐,” no statistical significance; *p < 0.05.

We merged the microarray data from GSE17048 and GSE61240, removing batch effects between the two datasets (Figure 6A–C), resulting in a comprehensive dataset consisting of 45 healthy individuals and 649 MS patients (586 RRMS, 43 PPMS, and 20 SPMS). Using the LIMMA method, 2195 DEGs were identified and presented in volcano plots (Figure 6D) and heatmaps (Figure 6E). Among the various differentially expressed inflammatory factors, CD40 is downregulated in the peripheral blood of MS patients (Figure 6F,G), particularly in those with RRMS (Figure 6H).

### 3.6 Consensus Clustering Analysis of RRMS Patients

**FIGURE 7:**
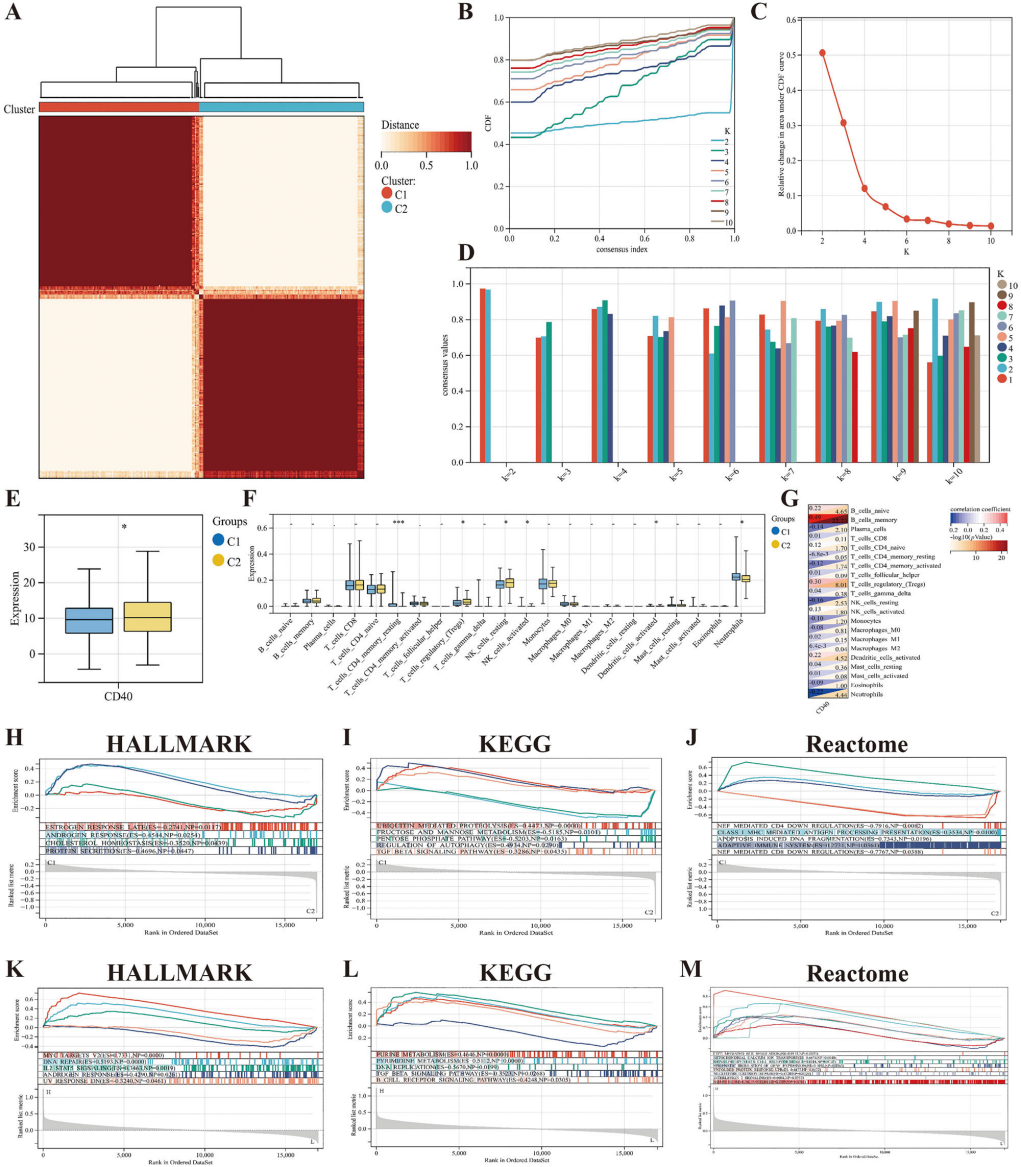
Identification, intergroup comparison, and functional analysis of CD40‐related RRMS subgroups. (A) RRMS patients are divided into two subgroups. (B–D) CDF curve (B), area under the cumulative distribution curve (C), and sample clustering consistency score (D) for consensus clustering analysis. (E, F) Levels of CD40 (E) and 22 types of immune cells (F) among subgroups. (G) Correlation matrix between CD40 and immune cells. (H–J) Intergroup GSEA analysis based on Hallmark (H), KEGG (I), and Reactome (J) pathways. A positive/negative ES indicates significant enrichment of the pathway in the C1/C2 subgroup, respectively. (K–M) Single‐GSEA analysis based on Hallmark (K), KEGG (L), and Reactome (M) pathways. A positive/negative ES reflects pathways that are significantly upregulated/downregulated by CD40‐mediated mechanisms in RRMS. “‐,” no statistical significance; *p < 0.05, ***p < 0.001.

The clustering analysis of 586 RRMS patients showed the optimal clustering effect when the number of clusters was *K* = 2 (C1 = 291, C2 = 295), with the sample clustering heatmap displaying the smallest intragroup differences (Figure 7A). Additionally, the CDF curve exhibited the smallest fluctuation range (Figure 7B), the largest area under the CDF curve (Figure 7C), and the highest clustering consistency score between the two subgroups (Figure 7D).

### 3.7 Intergroup Comparison After Cluster Analysis

Among the two RRMS subgroups, the CD40 level in C2 was higher than in C1, indicating a relatively better prognosis for RRMS patients in C2 (Figure 7E). In C1, the levels of resting memory CD4^+^ T cells, activated NK cells, and neutrophils were higher, whereas in C2, the levels of Tregs, resting NK cells, and activated dendritic cells were higher (Figure 7F). Additionally, CD40 was significantly positively correlated with memory B cells and significantly negatively correlated with neutrophils and resting NK cells (Figure 7G). Therefore, CD40 may play a key role in regulating the immune function of RRMS patients.

The GSEA method based on Hallmark, KEGG, and Reactome pathways was used to evaluate the functional differences between C1 and C2. In C1, ubiquitin‐mediated protein hydrolysis, autophagy regulation, major histocompatibility complex (MHC)‐mediated antigen processing and presentation, apoptosis‐induced DNA fragmentation, and adaptive immune regulation, as well as the TGF‐β signaling pathway, were significantly enhanced. In contrast, in C2, cholesterol homeostasis, fructose, mannose, and pentose phosphate metabolic pathways, as well as CD4 and CD8 downregulation pathways, were upregulated (Figure 7H–J). The concentration of CD40 may affect the involvement of these pathways in C1 and C2, ultimately leading to different symptoms and prognoses.

### 3.8 GSEA of CD40

The Single‐GSEA results indicate that CD40 primarily upregulates processes such as purine and pyrimidine metabolism, DNA repair, CD22‐mediated BCR regulation, mitochondrial calcium ion transport, unfolded protein response (UPR), and adaptive immune regulation in RRMS patients. However, CD40 downregulates the androgen response pathway and TGF‐β signaling pathway involved in the pathological process of RRMS (Figure 7K–M).

## 4 Discussion

Inflammation is a physiological response of the host to endogenous or exogenous infection or injury, coordinated by a complex network of cells and mediators, including circulating proteins such as cytokines and soluble receptors. However, dysregulation of the inflammatory pathway can lead to tissue damage and contribute to the progression of NDDs (Michaud et al. 2013). Numerous observational studies have preliminarily revealed the relationship between circulating inflammatory markers and NDDs, but very few MR analyses have clarified the causal relationship between the two. Existing MR studies have limitations, such as including only a single NDD and a small number of inflammatory proteins, conducting only unidirectional MR studies, results not corrected by FDR, and not combining multi‐omics analysis. For the first time, this study used bidirectional two‐sample MR analysis to evaluate the causal relationship between 91 circulating inflammatory proteins and 5 NDDs and combined transcriptomic techniques to validate the MR results.

The MR results suggest that an increase in CD40 protein levels is associated with a reduced risk of MS. MS is a chronic inflammatory demyelinating disease of the central nervous system (CNS), with 85% of cases being RRMS, which is the most common cause of nontraumatic neurological disability in young patients (Hemmer et al. 2015). The pathogenesis of MS is still unclear, characterized by dynamic inflammatory lesions caused by various activated immune cells such as T cells, B cells, monocytes, and macrophages in the CNS, ultimately leading to neurological deficits (Frohman et al. 2006). CD40 is a membrane‐bound co‐stimulatory protein that typically binds to its classic ligand CD40L to form a dimer. Its growing recognition as a therapeutic target for MS underscores its clinical significance. CD40–CD40L is widely expressed in blood immune cells, inflammatory cells infiltrating the CNS, and demyelinating plaques in the brains of MS patients (Ots et al. 2022). Elevated CD40–CD40L levels in the peripheral blood of MS patients can directly activate various immune cells, resulting in myelin degradation, axonal damage, and blood‐brain barrier disruption. Targeting the CD40–CD40L pathway demonstrates promising therapeutic potential for MS, though several clinical challenges remain to be addressed. The development of the anti‐CD40L monoclonal antibody IDEC‐131 was discontinued following reports of severe thromboembolic events and immunosuppression in clinical trials (Aarts et al. 2017). In contrast, the second‐generation anti‐CD40L antibody frexalimab demonstrated significant efficacy in reducing inflammatory brain lesions in MS patients, although mild‐to‐moderate adverse events, including headache and viral infections, were observed (Vermersch et al. 2024). The small‐molecule inhibitor 6877002 specifically blocks CD40‐TRAF6 interactions in experimental autoimmune encephalomyelitis (EAE) animal models, effectively alleviating neuroinflammation without inducing significant immunosuppression (Aarts et al. 2017). This finding suggests a promising new direction for clinical translation. To address the efficacy and safety limitations of current therapies, researchers have developed multiple strategies targeting CD40 in MS treatment, including precision drug delivery systems employing CD40L‐specific peptide ligands such as A25, combination therapies pairing CD40 inhibitors with immunomodulators, and targeted modulation of CD40‐mediated signaling pathways, including CD40‐NF‐κB. These approaches collectively enhance anti‐inflammatory effects while promoting neural repair, highlighting CD40's significant potential as a therapeutic target in MS (Aarts et al. 2017, D. Chen et al. 2016, Fadul et al. 2021). Although CD40‐targeted therapy for MS requires further large‐scale clinical trials to validate its long‐term efficacy, its dual capacity to modulate immune dysregulation and attenuate neuroinflammation offers promising therapeutic potential for MS patients.

Meanwhile, studies have also reported that CD40 may be protective in MS, supporting the MR results. A cross‐sectional study using flow cytometry to measure peripheral blood monocyte subsets in MS patients showed significant downregulation of CD40 expression on monocytes (Gjelstrup et al. 2018). Several GWAS studies have found that SNPs in the *CD40* gene, rs1883832C>T and rs6074022T>C, are associated with a higher risk of MS and induce a decrease in CD40 mRNA expression levels in whole blood (Gandhi et al. 2010; Wagner et al. 2015). Additionally, these risk sites reduce the expression level of CD40 protein on the surface of B cells, dendritic cells, and monocytes (Field et al. 2015). In the MS mouse model, CD40L deficiency exacerbates striatal lesions, neuronal demyelination, and meningitis in mice infected with Theiler's murine encephalitis virus (TMEV) (Drescher et al. 2000). In the absence of CD40, T cells directly enter the CNS of EAE mice and worsen the brain pathological phenotype (Abromson‐Leeman et al. 2001), demonstrating the protective potential of CD40–CD40L in preventing demyelinating diseases, promoting spontaneous myelination regeneration, and regulating immune responses.

The eQTLs of SNPs are mainly related to HSV‐1 infection, bile acid metabolism, and bile salt biosynthesis. HSV‐1 is a neurotropic virus that often resides in nerve cells and is associated with the demyelination and neurodegeneration processes of MS. CD40 activation can directly inhibit HSV‐1 proliferation (Vlahava et al. 2015; Bello‐Morales et al. 2021). Both MS patients and mouse models exhibit changes in gut microbiota, disruption of intestinal barriers, and decreased levels of bile acid metabolites. Fecal microbiota transplantation and bile acid supplementation are promising treatment options (Bhargava et al. 2020; Camara‐Lemarroy et al. 2018). Meanwhile, targeting the gut microbiota to mitigate anti‐CD40‐induced immunosuppressive toxicity highlights the potential role of CD40 in MS pathology by regulating bile acid metabolism (Blake et al. 2021).

CD40 is downregulated in the peripheral blood gene expression of MS, particularly in RRMS, consistent with the findings of Gjelstrup et al. (2018) and Marsh‐Wakefield et al. (2022). The infiltration levels of immune cells differ between the two RRMS subgroups. CD4^+^ T cells, activated NK cells, and neutrophils are increased in C1, while Tregs, resting NK cells, and activated dendritic cells are increased in C2. During the immune activation phase of MS, CD4^+^ T cells express CD40L, interacting with CD40 and causing a large secretion of inflammatory cytokines, which drives the rapid progression of MS neuroinflammation (Aarts et al. 2017). The RRMS single‐cell transcriptome is characterized by an increase in cytotoxic NK cells, and a higher neutrophil‐to‐lymphocyte ratio is associated with the severity and progression of MS (J. Liu et al. 2021; Aliyu et al. 2024). Tregs play a crucial role in maintaining autoimmune tolerance, limiting excessive inflammation, and tissue repair; their impaired function is considered a potential pathogenic mechanism of RRMS (Raϊch‐Regué et al. 2012). Additionally, inducing autoimmune tolerance via dendritic cells is an immunotherapy strategy for RRMS (Azimi et al. 2018). The changes in these immune cells can be used to monitor the disease activity and prognosis of CD40‐related MS.

GSEA revealed that processes such as MHC‐mediated antigen presentation, apoptosis, TGF‐β signaling pathways, ubiquitin‐mediated protein hydrolysis, and autophagy regulation were upregulated in C1, while cholesterol homeostasis, glucose metabolism, and low CD4/CD8 expression were upregulated in C2. CD40 can act as a co‐stimulatory molecule for MHC‐II to regulate the inflammatory cytokine network in MS, and inhibiting MHC‐II expression can alter the clinical outcome of MS (O'Keefe et al. 2002, Tang et al. 2021). Decreased CD40 expression in endothelial cells increases TGF‐β‐induced endothelial–mesenchymal transition (EndoMT), which can disrupt the blood‐brain barrier and is a marker found in MS autopsy samples (Takahashi et al. 2024; Mey and DeSilva 2022). CD40 orchestrates ubiquitin‐mediated proteolysis by recruiting E3 ubiquitin ligases (Hrd1 and NEDD4) to degrade target proteins (Blimp‐1, TRAF3), thereby modulating B‐cell differentiation and immune responses (Basu et al. 2016; Fang et al. 2014). Concurrently, CD40 enhances autophagy flux via upregulation of Beclin‐1 and LC3‐II, promoting antigen presentation and pathogen clearance in immune cells (Shen et al. 2022; Watanabe and Tsubata 2009; Van Grol et al. 2013). Dysregulation of CD40 expression may disrupt immune homeostasis and autophagy, potentially exacerbating neuroinflammation and demyelination in MS. Normalizing cholesterol, glucose metabolism, and CD4/CD8 levels is beneficial for delaying the progression of RRMS (Martin‐Gutierrez et al. 2024; Hwang et al. 2022; Zhong et al. 2022). Single‐GSEA results showed that CD40 can promote processes such as purine and pyrimidine metabolism, DNA repair, mitochondrial calcium transport, UPR, and adaptive immune regulation in RRMS patients, all of which have therapeutic potential for MS (Amirinejad et al. 2021; Rzagalinski et al. 2019; Holman et al. 2020; Stone and Lin 2015; Racosta and Kimpinski 2016). Conversely, CD40 inhibits androgen response pathways and TGF‐β signaling pathways. A risk correlation analysis involving prostate cancer patients undergoing androgen deprivation therapy (ADT) and those not receiving ADT showed that ADT significantly reduced the risk of developing autoimmune diseases, including MS (J.‐M. Liu et al. 2019). Elevated serum TGF‐β1 levels in RRMS patients (Nicoletti et al. 1998) and inhibition of TGF‐β1 can reduce Th17 cell infiltration in the CNS of mice with myelin oligodendrocyte glycoprotein (MOG) immune models, thereby alleviating disease severity (Luo et al. 2007). The exact mechanism of CD40's protective effects in MS remains to be further studied.

Our research has several advantages. First, all datasets are sourced from the latest and largest GWAS, ensuring the credibility of causal relationships. Second, bidirectional MR designs effectively reduce the impact of confounding factors. Third, this study includes 5 major NDDs and 91 circulating inflammatory proteins, making it the most comprehensive MR study to investigate their causal relationships. Fourth, we set strict screening criteria, such as correcting *p* values using the FDR method and retaining only MR results with a number of SNPs ≥ 10. Fifthly, we integrated transcriptome sequencing data to validate the protective effect of CD40 on MS and explored the underlying molecular mechanisms. However, this study also has certain limitations. The exclusive use of European‐ancestry data may limit the generalizability of findings, as allele frequencies and LD patterns of CD40/CD40L‐associated SNPs likely differ substantially in non‐European populations (e.g., African or East Asian), potentially compromising the effectiveness of IVs. To address this, trans‐ethnic MR analyses could validate result generalizability by integrating multiethnic GWAS data (East Asian, African, and Latin American populations) to evaluate causal association consistency and performing population‐stratified analyses to quantify effect‐size heterogeneity. Besides, this study relied exclusively on brain tissue eQTL data. Given that NDDs may involve complex crosstalk between the central nervous and peripheral immune systems (Zang et al. 2022), this single‐tissue approach potentially overlooks contributions from peripheral inflammatory mediators. Future studies should incorporate multi‐tissue expression databases (e.g., GTEx) to systematically evaluate CD40/CD40L expression patterns across tissues and their associations with MS, enabling more comprehensive genotype–phenotype analyses. Finally, we relaxed the threshold of *p* value, which means the correlation between exposure and IVs is relatively small, although the *F*‐statistics of all IVs are > 10.

## 5 Conclusions

Our MR analyses suggested that circulating CD40 protein is associated with a low risk of MS, with no statistical evidence supporting a bidirectional causal relationship between circulating inflammatory proteins and PD, AD, ALS, or IS. The eQTL analysis results indicate that HSV‐1, bile acids, and their metabolites may be downstream targets of CD40 intervention in MS. Bioinformatics analysis indicates that CD40 is significantly reduced in the peripheral blood of RRMS patients, with various immune cells, BP, and signaling pathways potentially playing a role. In summary, this study enhances the evidence supporting CD40's protective role in MS. Future investigations should further elucidate the pathophysiological mechanisms underlying CD40‐targeted therapies in MS, facilitating the development of individualized treatment approaches and novel therapeutic options for MS patients.

## Author Contributions

**Ziwei Gong**: data curation, formal analysis, methodology, software, visualization, writing ‐ original draft. **Rong Cao**: methodology, validation, software, formal analysis, data curation, writing ‐ original draft. **Hong Zhu**: data curation, software, supervision, validation, writing ‐ review and editing.

## Ethics Statement

All research data were derived from published GWAS or transcriptomic studies, and participants had signed informed consent forms in the original studies. The original dataset used in this study had obtained ethical approval from the ethics committees of various institutions. Therefore, further ethical review was not required.

## Conflicts of Interest

The authors declare no conflicts of interest.

### Peer Review

The peer review history for this article is available at https://publons.com/publon/10.1002/brb3.70586

## Supporting information



Supporting Information

Supporting Information

Supporting Information

Supporting Information

Supporting Information

Supporting Information

Supporting Information

Supporting Information

Supporting Information

## Data Availability

All datasets used in this study are available from the GWAS Catalog (https://www.ebi.ac.uk/gwas/), the IEU Open GWAS Project (https://gwas.mrcieu.ac.uk/), and the Gene Expression Omnibus (GEO, https://www.ncbi.nlm.nih.gov/geo/). Additionally, other data derived from analyses in this study are available from the corresponding authors upon reasonable request.
