# Supplementary figures and images for: Exploring the Causal Association Between 91 Circulating Inflammatory Proteins and Neurodegenerative Diseases: A Bidirectional Two‐Sample Mendelian Randomization and Bioinformatics Analysis
Source: Brain Behav. 2025 Jun 4;15(6):e70586. doi: 10.1002/brb3.70586 (PMC12134489; doi:10.1002/brb3.70586)

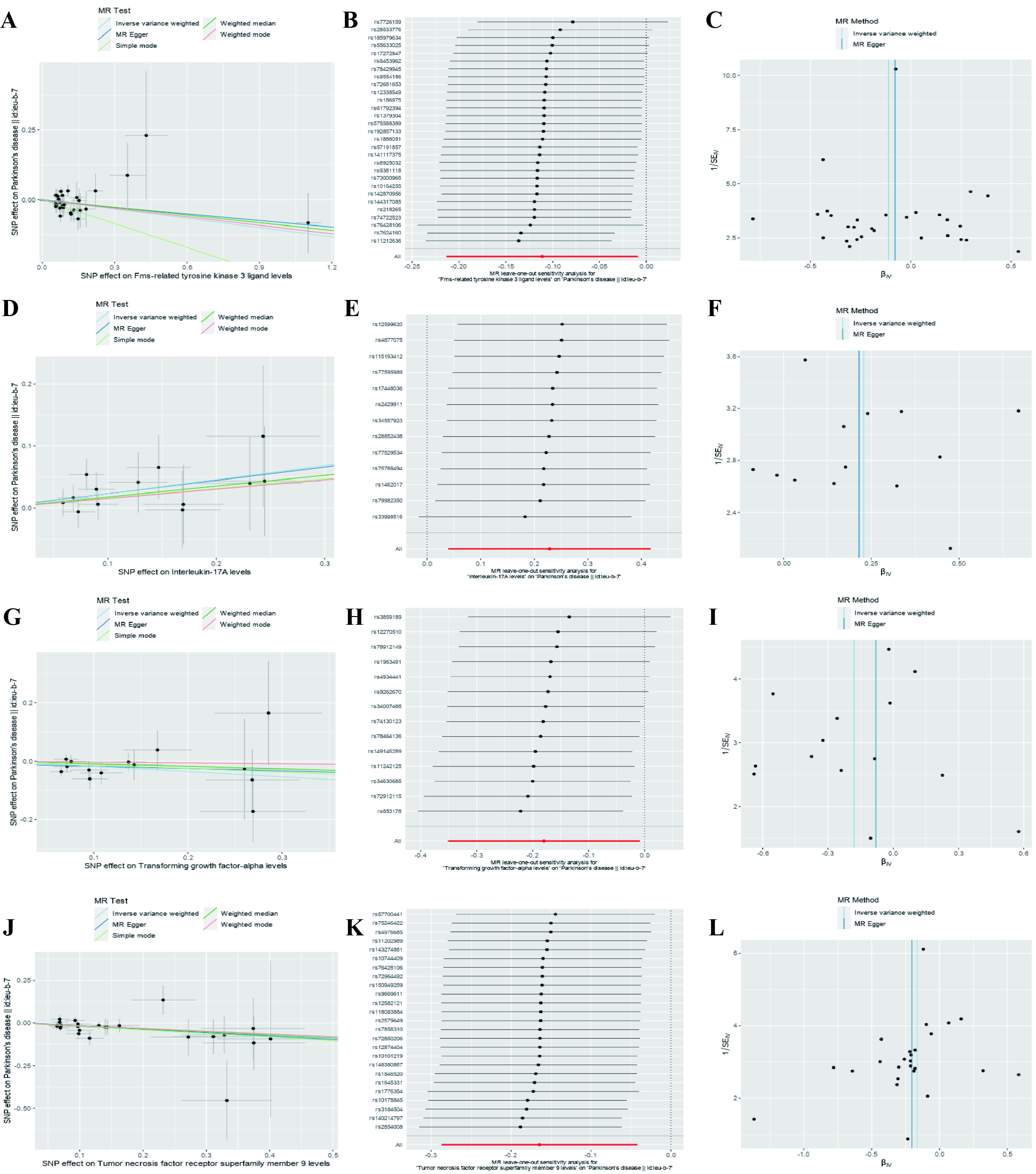

Supplement: Supplementary file 1 — Supporting Information [file BRB3-15-e70586-s009.jpg]

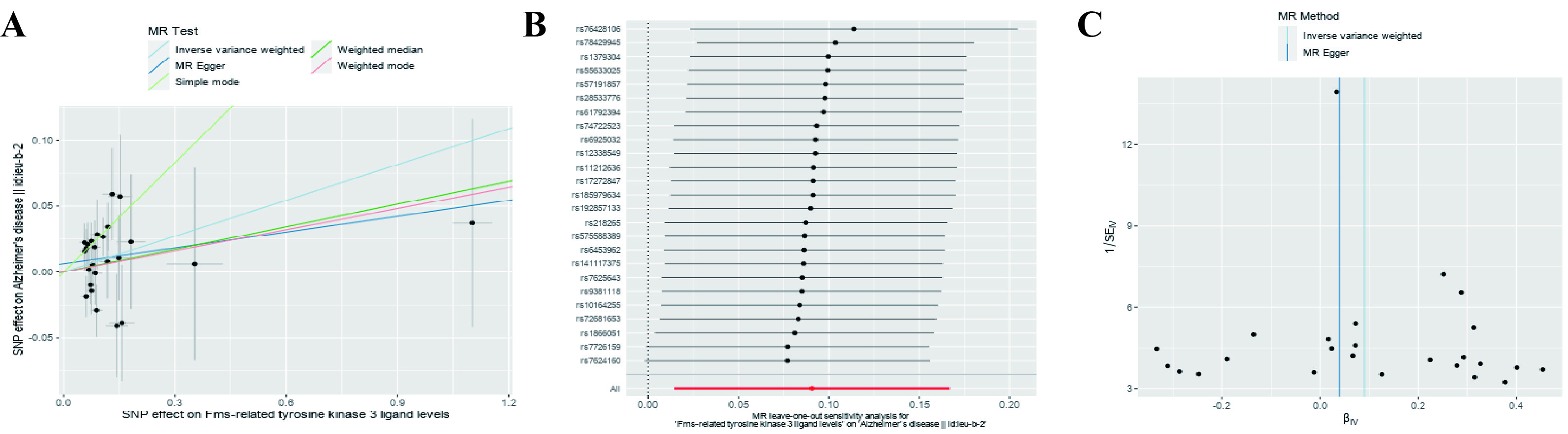

Supplement: Supplementary file 2 — Supporting Information [file BRB3-15-e70586-s006.jpg]

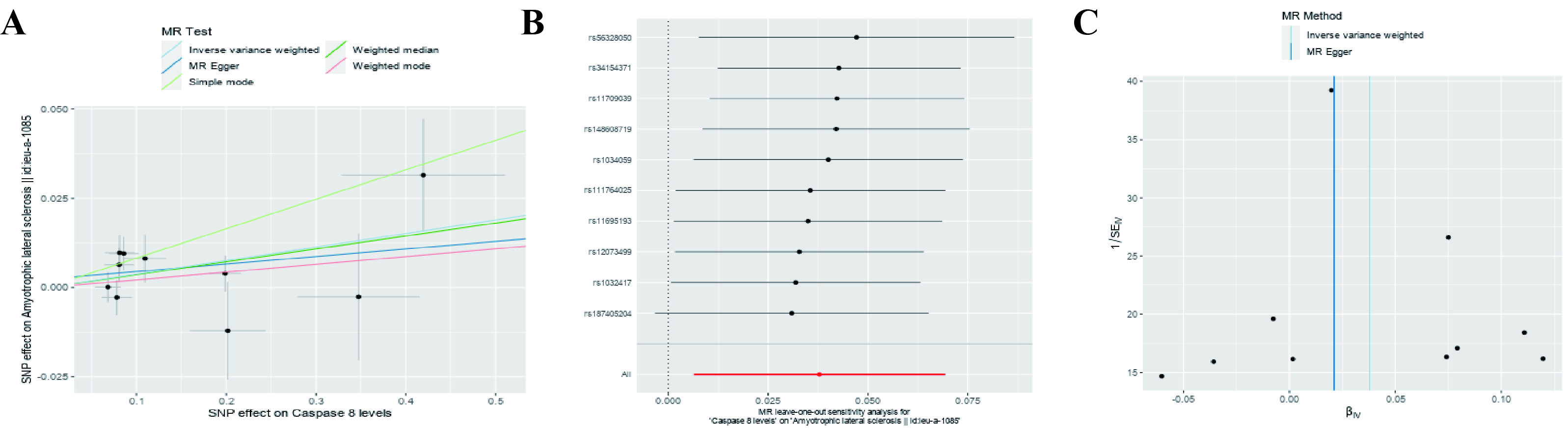

Supplement: Supplementary file 3 — Supporting Information [file BRB3-15-e70586-s002.jpg]

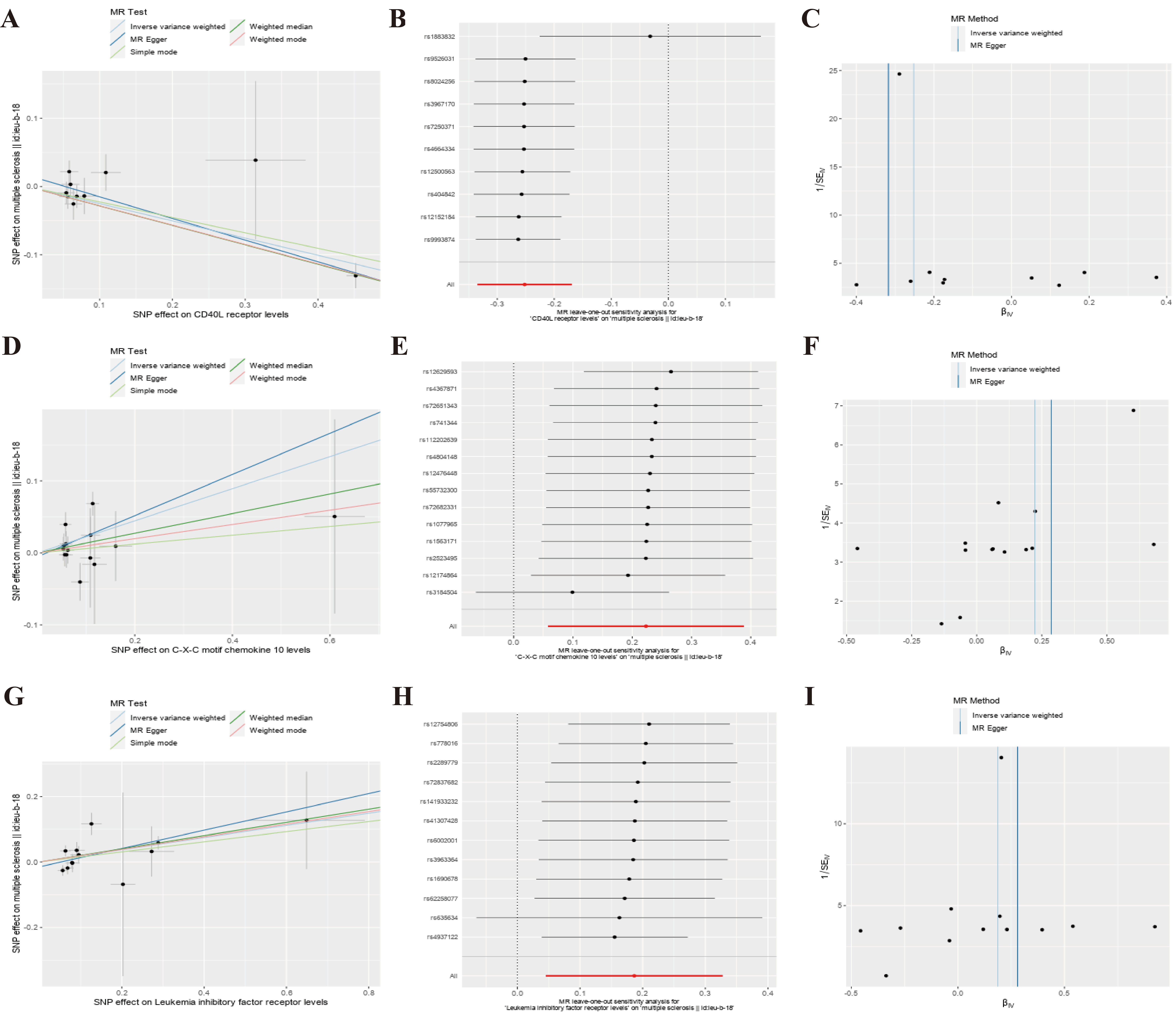

Supplement: Supplementary file 4 — Supporting Information [file BRB3-15-e70586-s001.jpg]

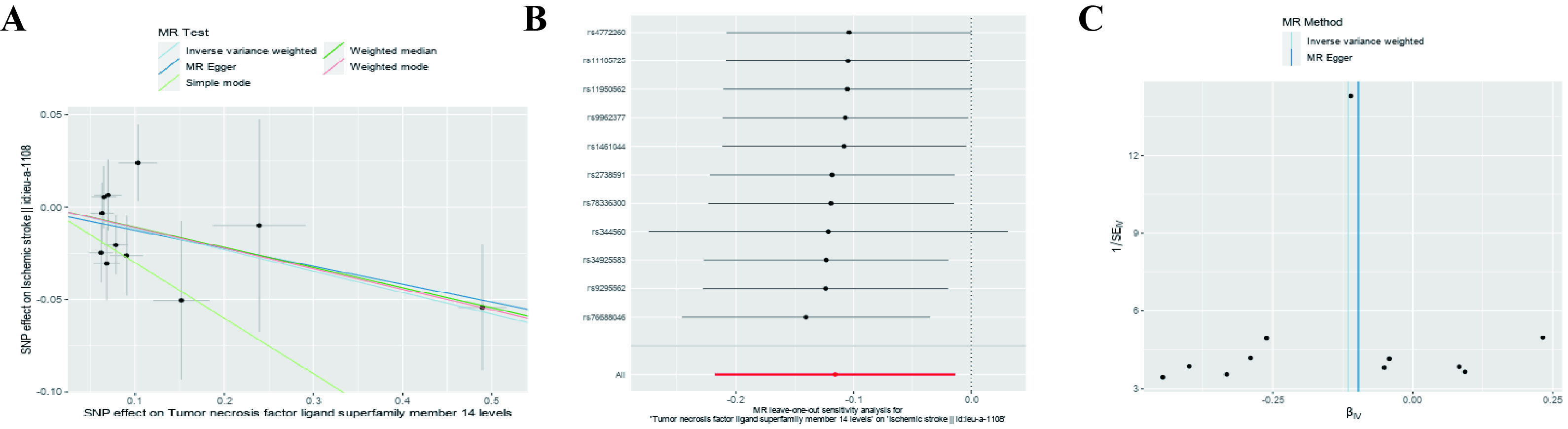

Supplement: Supplementary file 5 — Supporting Information [file BRB3-15-e70586-s003.jpg]

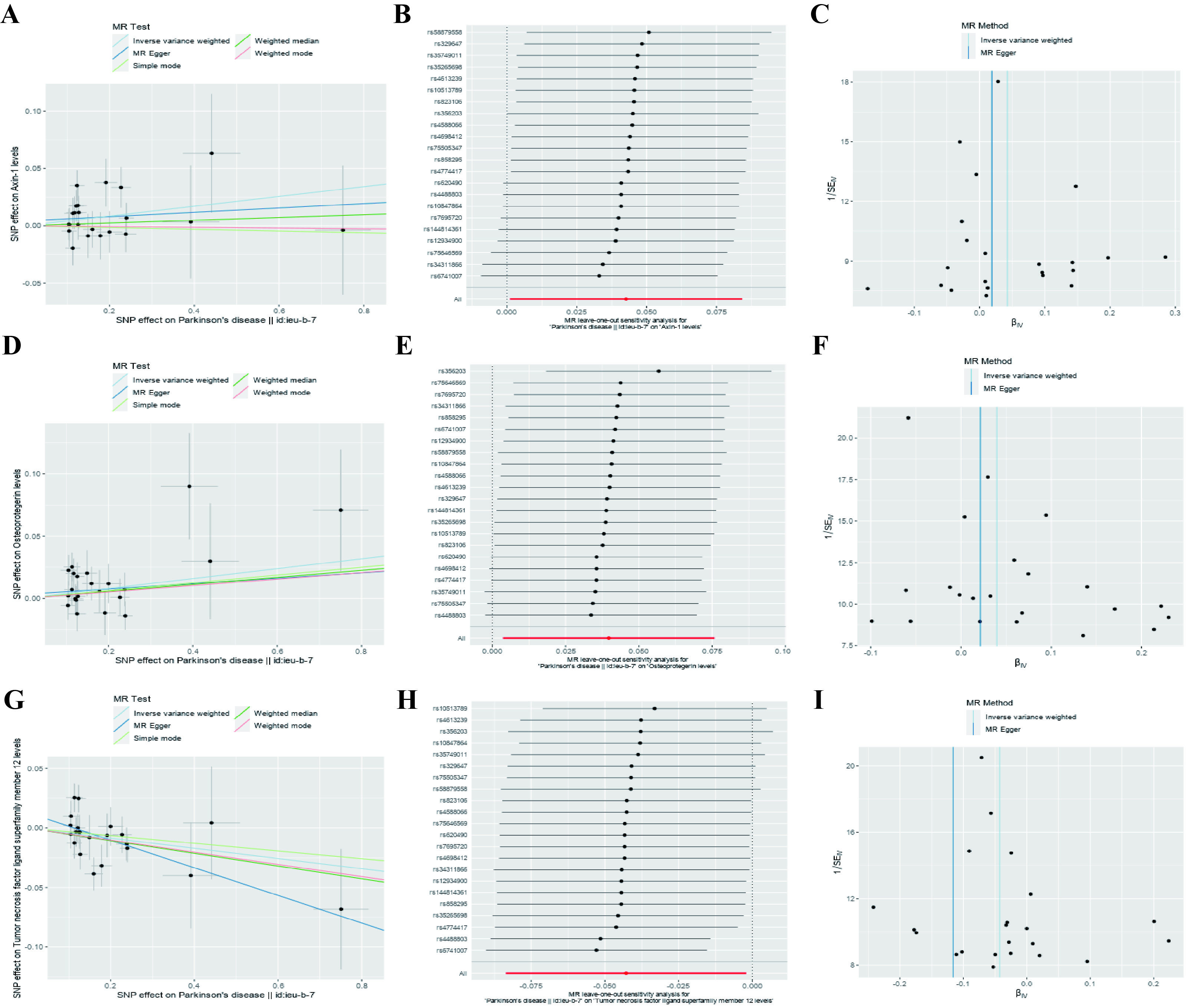

Supplement: Supplementary file 6 — Supporting Information [file BRB3-15-e70586-s005.jpg]

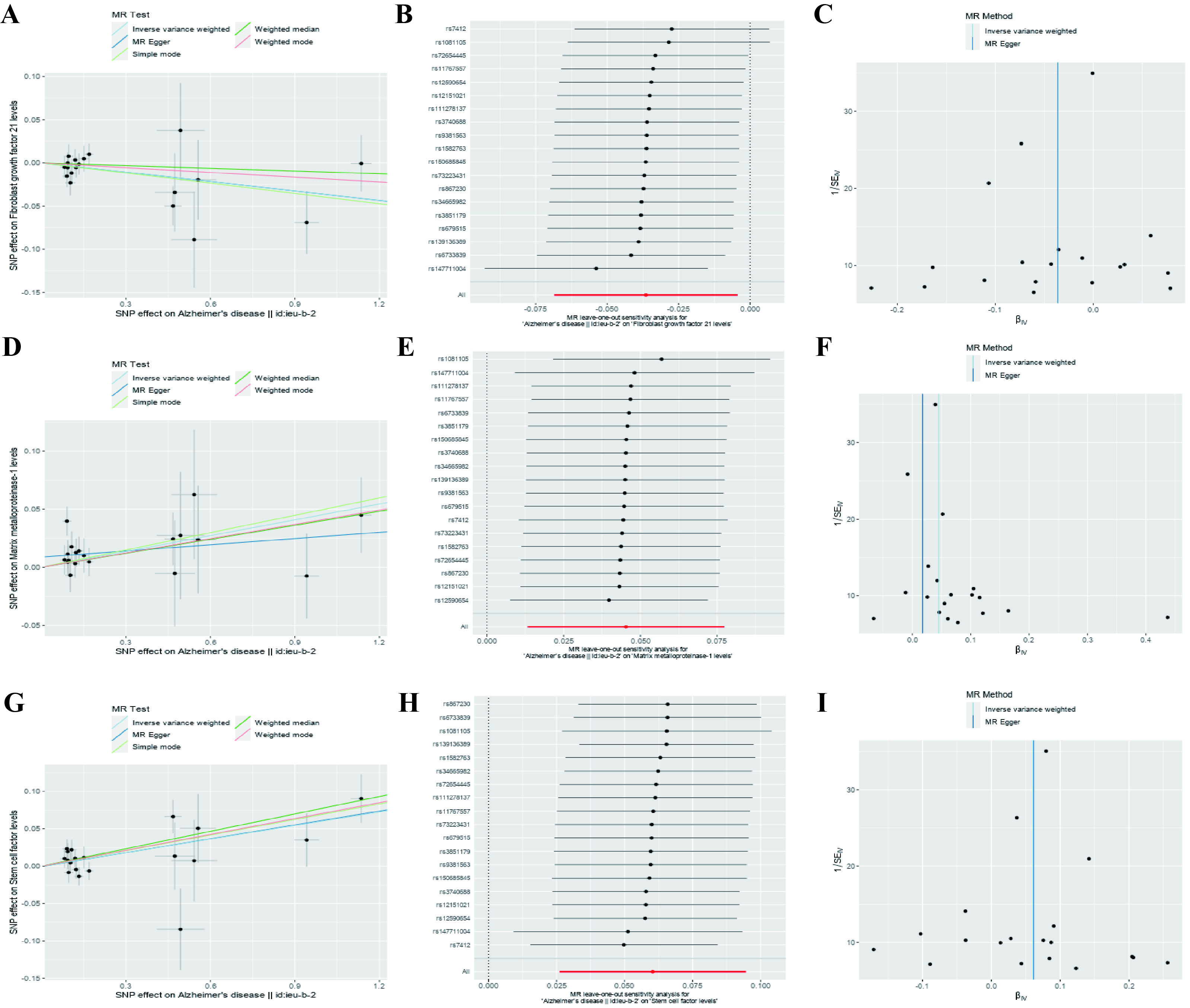

Supplement: Supplementary file 7 — Supporting Information [file BRB3-15-e70586-s007.jpg]
