# Supplementary material for: Exploring the Causal Association Between 91 Circulating Inflammatory Proteins and Neurodegenerative Diseases: A Bidirectional Two‐Sample Mendelian Randomization and Bioinformatics Analysis
Source: Brain Behav. 2025 Jun 4;15(6):e70586. doi: 10.1002/brb3.70586 (PMC12134489; doi:10.1002/brb3.70586)
Supplement: Supplementary file 9 — Supporting Information [file BRB3-15-e70586-s004.docx]

**Supplementary Figure 1
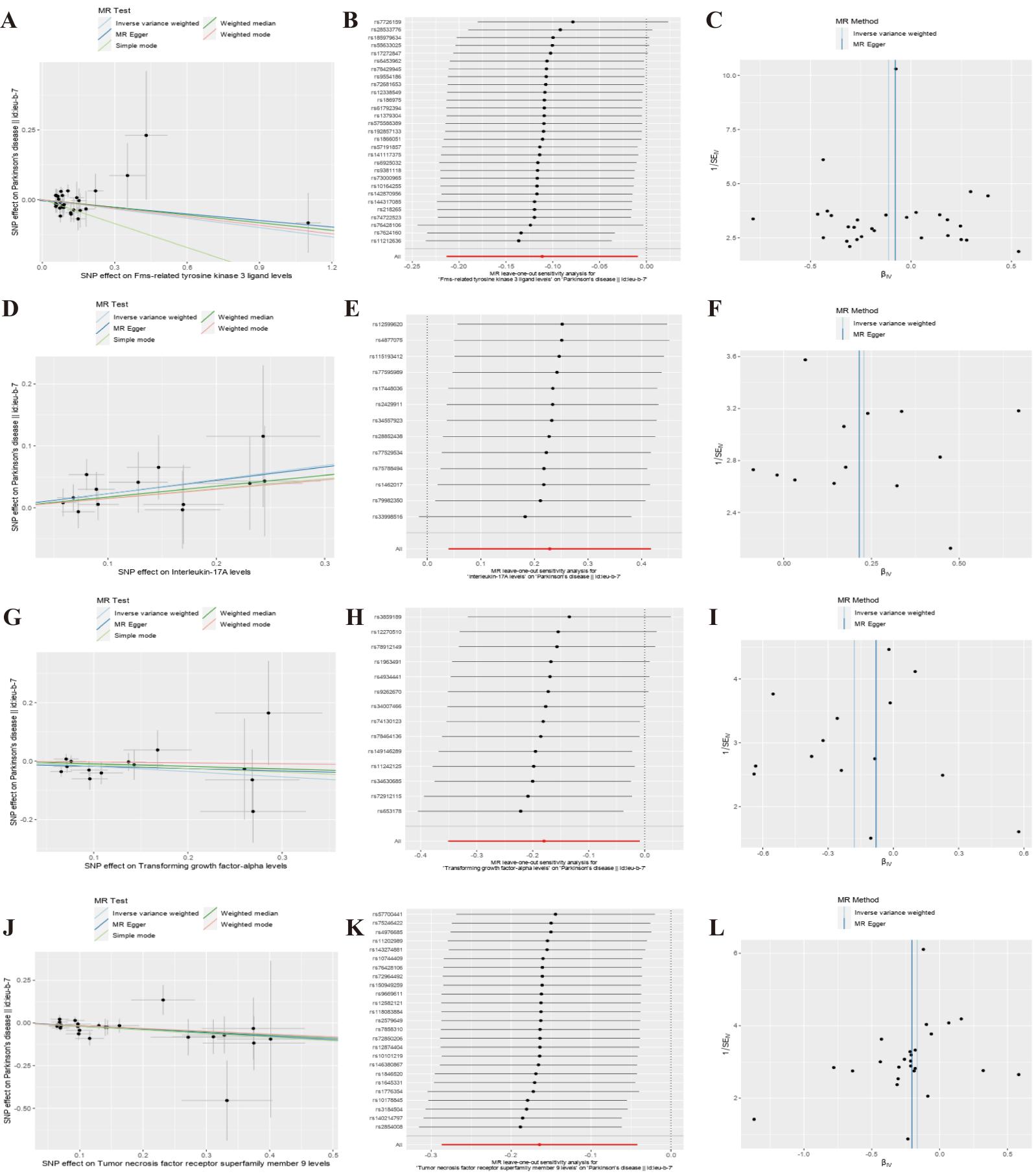
:**Flt-3L, IL-17A, TGF-α and TNFRSF9-associated SNPs with risk of PD. (A,D,G,J) Scatter plot of Flt-3L, IL-17A, TGF-α and TNFRSF9 with risk of PD. (B,E,H,K) MR leave-one-out sensitivity analysis for Flt-3L, IL-17A, TGF-α and TNFRSF9 on PD. (C,F,I,L) Funnel plot of Flt-3L, IL-17A, TGF-α and TNFRSF9 instruments strength on PD.


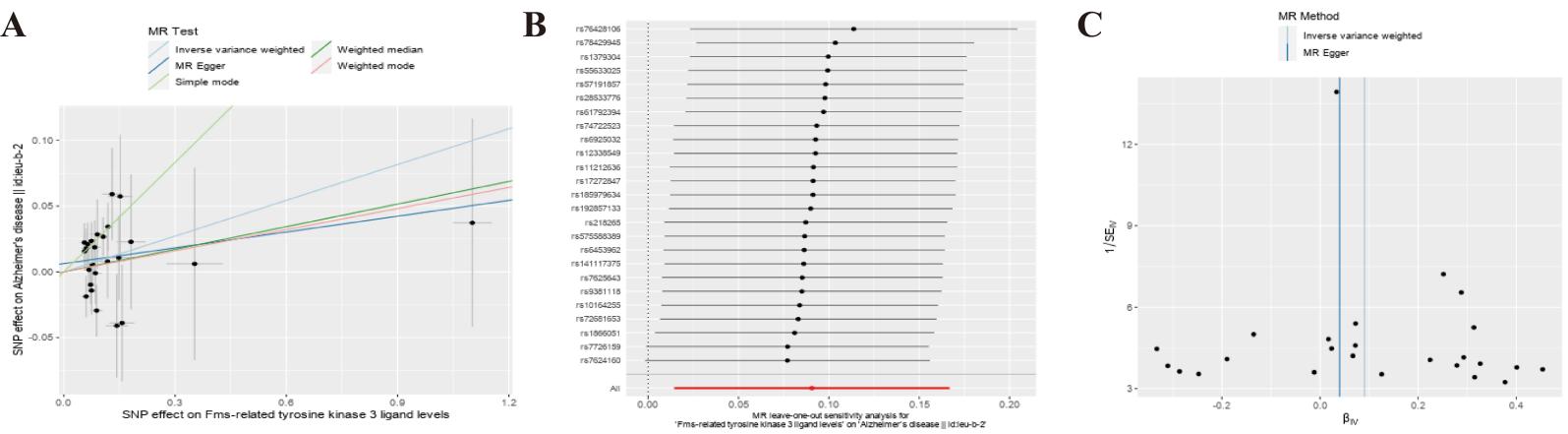
**Supplementary Figure 2:** Flt-3L-associated SNPs with risk of AD.(A) Scatter plot of Flt-3L with risk of AD. (B) MR leave-one-out sensitivity analysis for Flt-3L on AD. (C) Funnel plot of Flt-3L instruments strength on AD.


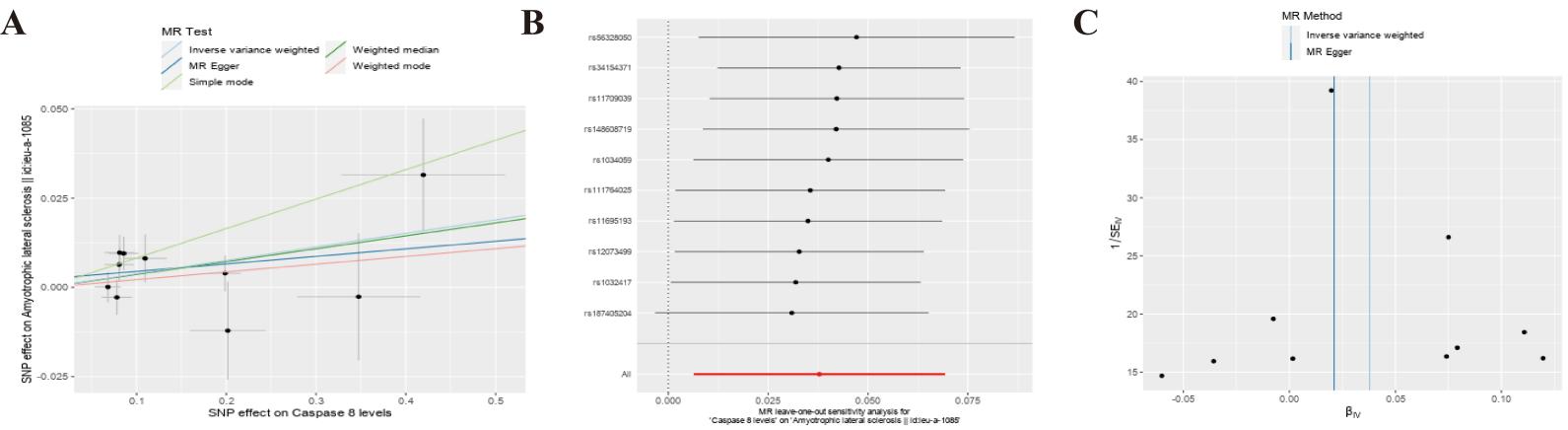


**Supplementary Figure 3:** Caspase-8-associated SNPs with risk of ALS.(A) Scatter plot of Caspase-8 with risk of ALS. (B) MR leave-one-out sensitivity analysis for Caspase-8 on ALS. (C) Funnel plot of Caspase-8 instruments strength on ALS.


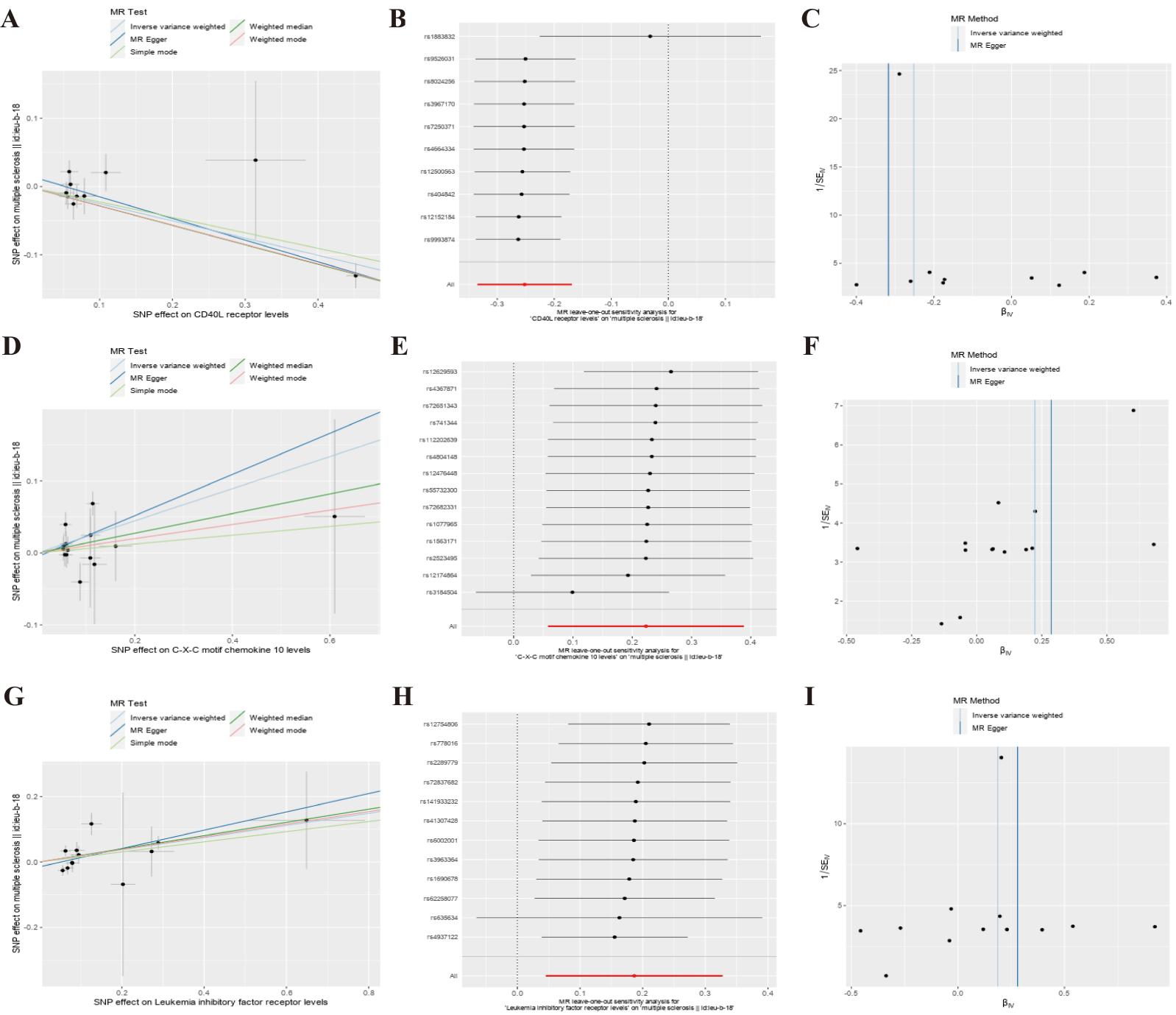
**Supplementary Figure 4:** CD40L receptor,CXCL10 and LIF receptor-associated SNPs with risk of MS.(A,D,G) Scatter plot of CD40L receptor,CXCL10 and LIF receptor with risk of MS. (B,E,H) MR leave-one-out sensitivity analysis for CD40L receptor,CXCL10 and LIF receptor on MS. (C,F,I) Funnel plot of CD40L receptor,CXCL10 and LIF receptor instruments strength on MS.


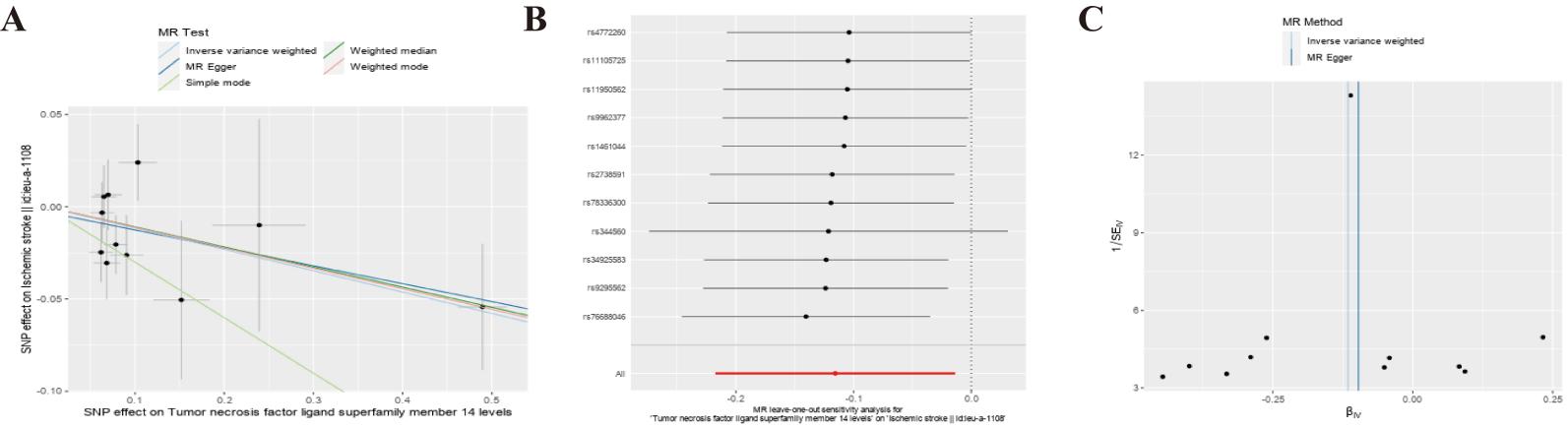
**Supplementary Figure 5:** TNFSF14-associated SNPs with risk of IS. (A) Scatter plot of TNFSF14 with risk of IS. (B) MR leave-one-out sensitivity analysis for TNFSF14 on IS. (C) Funnel plot of TNFSF14 instruments strength on IS.


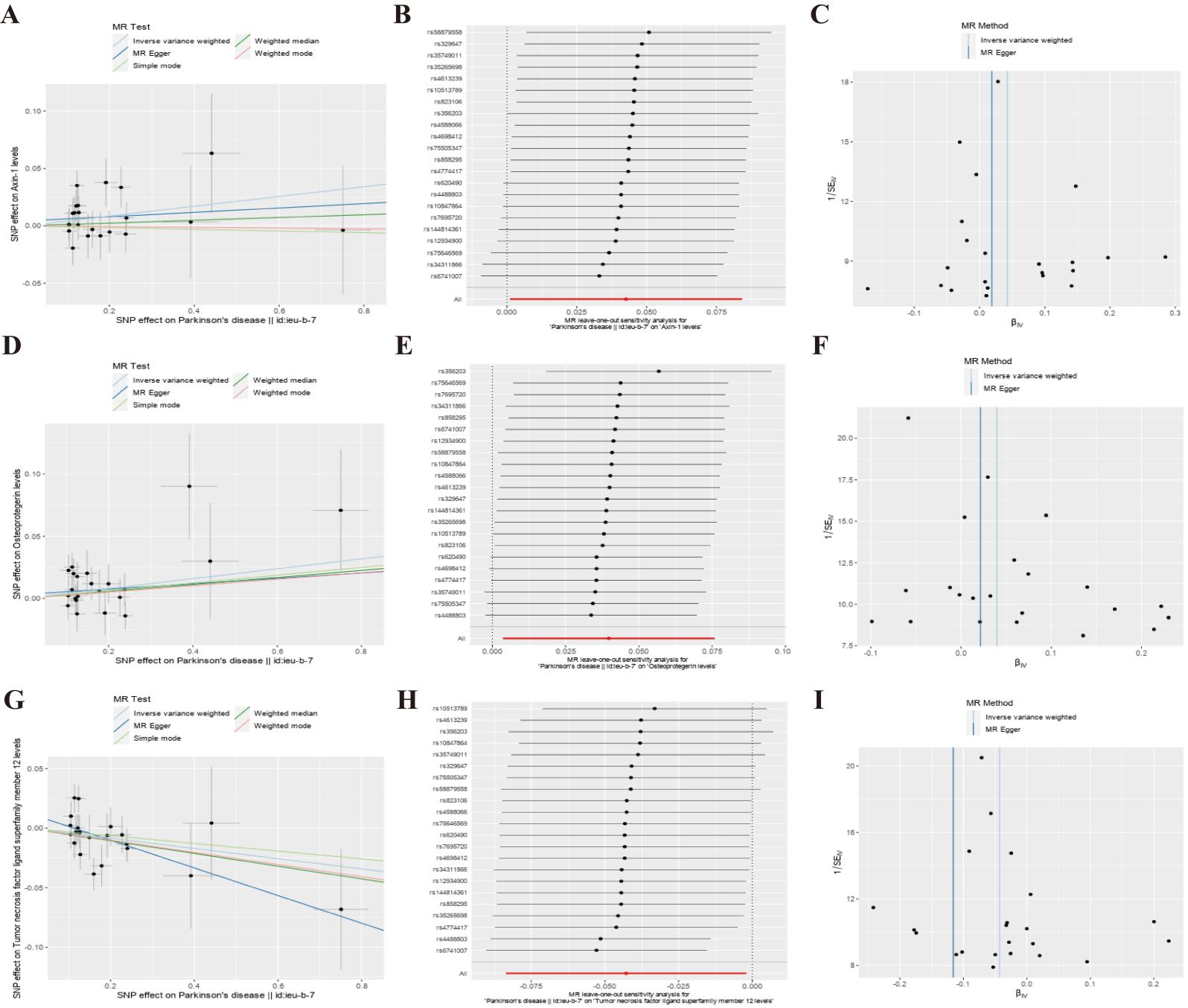
**Supplementary Figure 6:** PD-associated SNPs with Axin-1,OPG and TNFSF12. (A,D,G) Scatter plot of PD on Axin-1,OPG and TNFSF12. (B,E,H) MR leave-one-out sensitivity analysis for PD on Axin-1,OPG and TNFSF12. (C,F,I) Funnel plot of PD on Axin-1,OPG and TNFSF12.


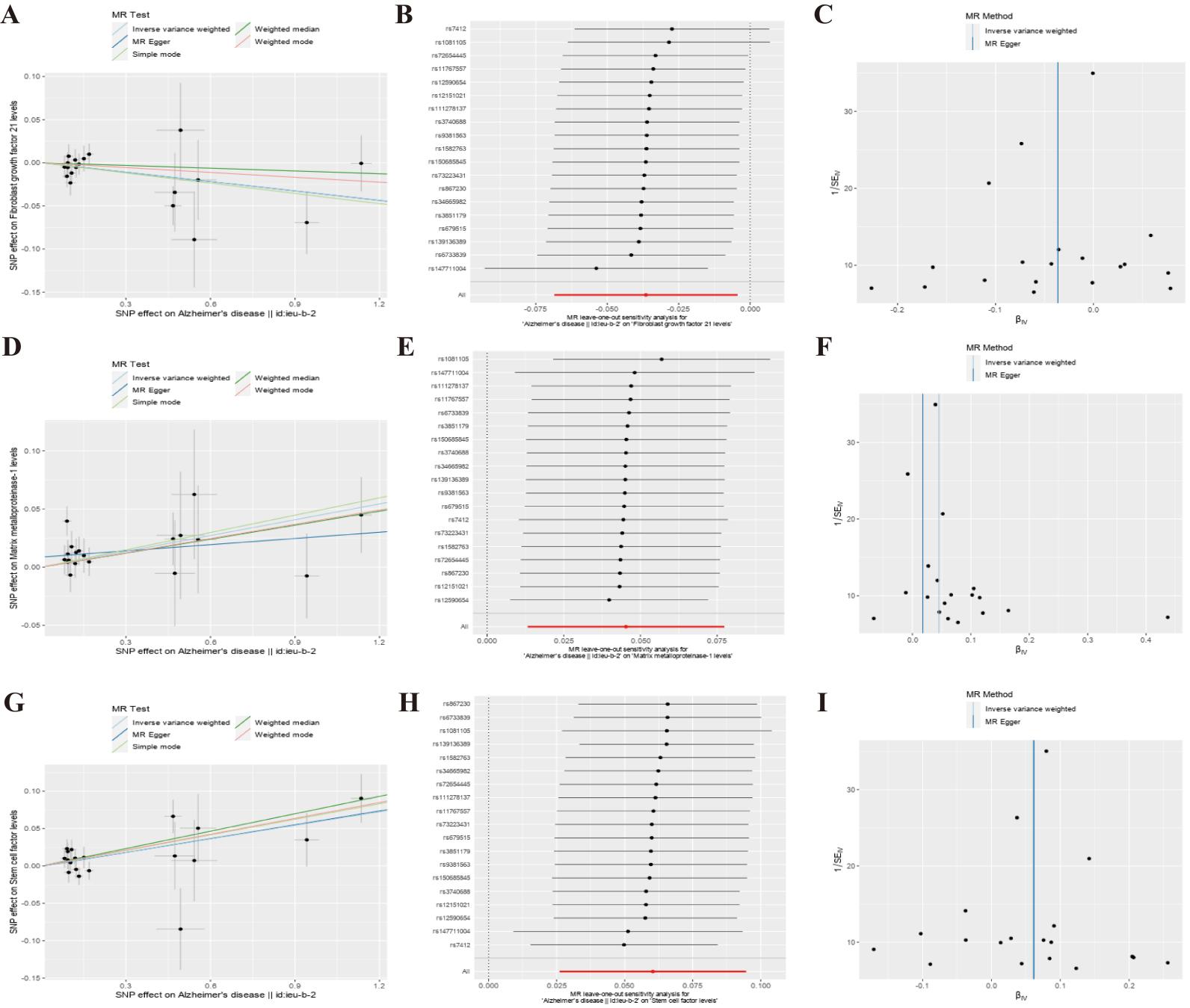
**Supplementary Figure 7:** AD-associated SNPs with FGF21,MMP-1 and SCF. (A,D,G) Scatter plot of AD on FGF21,MMP-1 and SCF. (B,E,H) MR leave-one-out sensitivity analysis for AD on FGF21,MMP-1 and SCF. (C,F,I) Funnel plot of AD on FGF21,MMP-1 and SCF.
